# Non-genomic Effects of Estrogen on Cell Homeostasis and Remodeling With Special Focus on Cardiac Ischemia/Reperfusion Injury

**DOI:** 10.3389/fendo.2019.00733

**Published:** 2019-10-25

**Authors:** Rossella Puglisi, Gianfranco Mattia, Alessandra Carè, Giuseppe Marano, Walter Malorni, Paola Matarrese

**Affiliations:** ^1^Center for Gender Specific Medicine, Istituto Superiore di Sanità, Rome, Italy; ^2^School of Medicine, University of Rome Tor Vergata, Rome, Italy

**Keywords:** estrogen, non-nuclear estrogen receptors, cardiomyocytes, vascular cells, ischemia/reperfusion, myocardial infarction, sex, gender

## Abstract

This review takes into consideration the main mechanisms involved in cellular remodeling following an ischemic injury, with special focus on the possible role played by non-genomic estrogen effects. Sex differences have also been considered. In fact, cardiac ischemic events induce damage to different cellular components of the heart, such as cardiomyocytes, vascular cells, endothelial cells, and cardiac fibroblasts. The ability of the cardiovascular system to counteract an ischemic insult is orchestrated by these cell types and is carried out thanks to a number of complex molecular pathways, including genomic (slow) or non-genomic (fast) effects of estrogen. These pathways are probably responsible for differences observed between the two sexes. Literature suggests that male and female hearts, and, more in general, cardiovascular system cells, show significant differences in many parameters under both physiological and pathological conditions. In particular, many experimental studies dealing with sex differences in the cardiovascular system suggest a higher ability of females to respond to environmental insults in comparison with males. For instance, as cells from females are more effective in counteracting the ischemia/reperfusion injury if compared with males, a role for estrogen in this sex disparity has been hypothesized. However, the possible involvement of estrogen-dependent non-genomic effects on the cardiovascular system is still under debate. Further experimental studies, including sex-specific studies, are needed in order to shed further light on this matter.

## Introduction

Cardiovascular diseases (CVD), including acute myocardial infarction (MI), represent leading causes of morbidity and mortality worldwide in both sexes. However, in past years, the risk of CVD was underestimated in women due to the mistaken belief that women could somehow be protected (1, 2). Although it was observed that women develop coronary artery disease about 10 years later than men, they show a higher prevalence of cardiovascular risk factors at the same times of their lifespan (3). Even currently CVD continue to be perceived as predominantly male pathologies, leaving women vulnerable to CVD due to an inadequate prevention. However, even if women in their reproductive age have a lower risk of cardiovascular events, their advantage decreases after menopause, so that CVD are the leading cause of death in women older than 65 years (4). In fact, in Europe, CVD cause a greater proportion of deaths among women than men (5, 6), also representing a critical economic burden (7).

The mechanisms leading to MI are due to a blocked blood flow resulting in various biochemical and metabolic alterations within the myocardium, i.e., in its main cell components: the cardiomyocytes (CMs). These cells undergo a series of well-characterized alterations, including mitochondrial dysfunction and, if prolonged, the death of cardiomyocytes (CMs). Obviously, ischemic events also induce damage in vascular cells and cardiac fibroblasts (CFs). The ability of cardiac tissue to recover after these events is carried out through a complex process of remodeling, orchestrated by CFs, inflammatory cells and cardiomyocytes (8). A number of complex cellular and molecular pathways, including antioxidant pathways and hormones, have been demonstrated to be able to counteract the damage. Imbalance or failure of these pathways leads to adverse remodeling of the heart and poor prognosis. However, the precise mechanisms of cardiomyocytes molecular injury after MI are still to be elucidated in detail (9). Some of these determinants are of interest of this work and are listed here below.

Following a MI, the left ventricle undergoes a remodeling that involves the removal of the necrotic tissue that is replaced by extracellular matrix proteins. The removal of necrotic tissue is carried out by the immune cells that polarize and release enzymes, such as matrix metalloproteinases (MMPs) and reactive oxygen species (ROS) (10). It was observed that infiltrating leukocytes release cytokines and growth factors such as pro-inflammatory interleukin IL-1β and reparative transforming growth factor β (11) that contribute to microenvironment alteration. This inflammatory state has been shown to be different in males and females either in animal models or in humans. In particular, females have a more moderate response to inflammatory stimuli; for example, in sepsis and atherosclerosis they have lower pro-inflammatory leukocyte-mediated inflammation and a faster resolution of inflammation compared with males (12, 13). Although it is known that XX cells have a more pronounced antioxidant capability (14–17), this matter should be better investigated in post-ischemic MI-associated damage.

It has been observed that early restoration of coronary blood flow after MI plays an important role in minimizing myocardial tissue injury through various types of therapy, such as thrombolytic therapy, coronary artery bypass grafting or primary percutaneous intervention (18). However, reperfusion may further contribute to newer myocardial damage defined as myocardial ischemia/reperfusion (I/R) injury, in which oxidative stress plays a critical role igniting ROS generation eventually leading to necrotic, apoptotic or autophagic cell death (19). Accordingly, current anti-apoptotic agents have generally been reported to safeguard the heart from I/R injury (20–22). However, increasing evidence also indicates that modulation of autophagy, that can be considered as a cytoprotective mechanism that leads to cell death only once all the energy supply derived by intracellular materials are exhausted, is now considered as a novel therapeutic strategy in myocardial I/R injury (23).

Although sex steroid hormones, particularly estrogens, appear to be involved through genomic and non-genomic effects in cell remodeling, molecular mechanisms remain still unknown (24, 25). Females undergo more efficient cardiac remodeling after ischemia/reperfusion injury most likely due to the cytoprotective effects of estrogen via an unknown mechanism. The regulatory effects of estrogen in cardiac sensitivity to I/R injury could have in fact many potential therapeutic implications, e.g., influencing strategies in acute coronary syndrome management. Tamargo and co-workers shed some light on this matter discussing in detail the efficacy and safety of several drugs of common use in cardiovascular diseases taking into account both sexes (6).

## Estrogen and Estrogen Receptors

Several estrogens, including estrone (E1), 17β-estradiol (E2), and estriol (E3) are present in the adult bloodstream, where E2 is the most represented and exerts many effects in both physiological and pathological conditions including cancer (26). In addition to its production in the ovaries of fertile women, E2 can be produced in other tissues as a product of enzymatic conversion of testosterone by aromatase (27). This enzyme is expressed in different extragonadic tissues, such as fat, bone and brain (28). Furthermore, increasing lines of evidence also demonstrate the local production of aromatase by heart and blood vessels of both sexes (29, 30).

E2 biological activities pass through its interaction with the estrogen receptors ERα and ERβ. Moreover, several polymorphisms that could be of relevance in CVD have been reported for these receptors (31–33). Initially identified into cytosol and nucleus, ERα and ERβ have more recently been described also at the level of the different intracellular compartments like endoplasmic reticulum, Golgi and mitochondria, other than plasma membrane (34, 35). Indeed, the different intracellular localization of these receptors impacts their specific signaling cascades and their ability to control cell growth, differentiation, survival or death (36–38). Besides ERα and β, an additional E2 binding responsive receptor, named G-protein-coupled estrogen receptor (GPER) has been identified (39). GPER is a member of the family of 7-transmembrane G protein-coupled receptors (GPCRs) and, besides plasma membrane, it has been localized in various intracellular organelles where it mediates several E2 effects (40).

### Signaling Pathways of Estrogen Receptors in Brief

Estrogen receptors transmit hormonal signals through three different pathways. The first one, known as “classic” or genomic, regulates the expression of target genes by DNA binding at specific response elements (EREs). Upon E2 binding, ERs dissociate from the complex formed with some heat shock proteins (like HSP70 and HSP90) in the cytosol, change their conformation and migrate as homo- or hetero-dimers into the nucleus (41).

**Figure 1:**
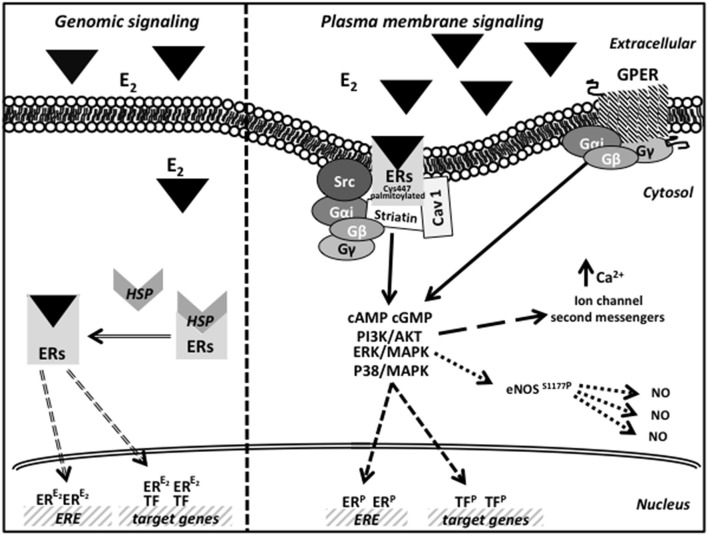
Schematic picture of estrogen action by genomic and plasma membrane ER/GPER signaling pathways.

The second signaling is controlled by an indirect ER binding to DNA, mediated by different co-factors (like SP-1, AP-1, and NF-κB) that exert their transcription regulation by physical interaction with DNA (42). Finally, in the non nuclear pathway, E2 induces very rapid cellular effects, acting through receptors localized at the cell membrane, cytoplasm, and mitochondria. Soon after binding E2, the membrane receptors interact with the Gα and Gβγ proteins to stimulate rapid signals (cAMP and cGMP) and trigger the activation of several transduction pathways (43, 44). The activation of kinases phosphorylates ER or other transcription factors resulting in gene expression regulation (45). As far as GPER is concerned, after E2 binding, it mediates a rapid membrane response involving the activation of kinases, ion channels and second messengers (46). In particular, in the endoplasmic reticulum, GPER activation induces calcium release and PI3K-Akt pathway activation, thus inducing cell proliferation (39, 40). Moreover, although still debated, it seems now clear that GPER does not physically associate with the mitochondria but, instead, its ability to regulate intracellular calcium levels indirectly affects mitochondrial function, including the so-called mitochondrial-induced cell death (47). Earliest studies on GPER also suggest how this receptor, although indirectly, regulates gene expression via an importin-dependent mechanism (48, 49). A schematic picture of possible estrogen action by genomic and plasma membrane ER/GPER signaling pathways is reported in Figure 1.

### Expression of Estrogen Receptors in the Cardiac Tissue

First evidence of ERα and ERβ expression in the cardiac tissue comes from a study performed in both female and male rat cardiac myocytes and fibroblasts (50). Subsequently, both the ERs were described in the human heart tissue (51) Later, ERα was localized in the nucleus and in the sarcolemma and intercalated discs of human cardiomyocytes (52). Additional data obtained from female and male mice hearts showed that ERα was mainly localized to the sarcolemma whereas ERβ to the nucleus and cytosol of the ventricular and atrial cells (53). ERβ was also described in human cardiac mitochondria (54). More recently, isolated mouse cardiomyocytes showed the presence of all the three ERα isoforms (ERα66, ERα46, and ERα36) in the nucleus (55). However, conflicting evidence also exists as concerns ERβ expression and localization in cardiomyocytes. Of course, the use of antibodies of doubtful specificity (56) as well as the wide variability of animal models and samples analyzed (e.g., whole heart or isolated cardiomyocyte lysates) caused the production of mutually contradictory data. For example, the presence of ERβ in human cardiac mitochondria (54) is still debated (57) and some reports have documented the total absence of ERβ in isolated cardiomyocytes (55). More recently, in a study exclusively conducted at mRNA level in rat cardiovascular tissues, high expression levels of ERα were detected, followed by GPER in terms of abundance, whereas ERβ appeared as nearly undetectable (58). Finally, in line with these results, the implication of ERβ in heart functional recovery after treatment with specific agonists in different animal models of heart failure also appears as still unclear (59–61).

As regards vascular smooth muscle cells (VSMCs), ERα was found to localize to the nuclei and to the plasma membrane in combination with caveolin-1, whereas ERβ was predominantly nuclear (62, 63). Both estrogen receptors have been described also in human adult aortic VSMCs (64). In these cells ERα and ERβ appear as localized at the level of caveolae where a direct binding to striatin is essential for their membrane localization (65). Also GPER was detected in numerous cardiac compartments of the human heart (66) and in coronary artery VSMCs (67). During myocardial hypoxia due to infarction, GPER seems to be upregulated in cardiomyocytes (68).

The question whether the beneficial actions offered by estrogen are due to ERα or to ERβ stimulated a large number of *in vivo* studies (69, 70). These studies were conducted in genetically modified mice and the use of selective agonists or antagonists of these receptors. However, which ER could play a major protective role against I/R injury is still under debate. In fact, a role either for ERα (71–74) or for ERβ (61, 75–77) has been hypothesized. This discrepancy could be due to different models of I/R and/or to different doses and timing of treatments taken into consideration.

### Estrogen Receptors: Genetically Modified Mice

As mentioned above, experimental studies involving animal models contributed to delineate the mechanisms involved in sex-related differences in cardiac tolerance to ischemia. In particular, most information derives from the study of genetically modified animals (see Table 1). Unfortunately, several studies have been performed almost exclusively on male animals, without taking into account the differences in hormonal fluctuations between sexes (127). In particular, studies based on different ERα gene targeting in murine models have defined the role specifically played by this receptor with particular reference to the different functional domains that compose the protein. As a matter of fact both estrogen receptors are composed by six functionally distinct protein regions like a DNA binding domain (DBD), a ligand-binding domain (LBD), a central region containing a nuclear localization sequence (NLS) and two regions acting as transcriptional activators (AF1 and AF-2), respectively located at the carboxy- and amino-terminal ends (128). The protein region responsible for the activity of E2 in the vascular system and in the metabolic function was identified in the AF2 domain (96), while the AF1 domain seems to be mainly involved in the reproductive function (98). In the same way, it was demonstrated that the localization at the plasma membrane of the receptor was closely dependent on its palmitoylation, which in turn favors its association with caveolin-1 in the lipid rafts (99, 100). Indeed, any mutation blocking one of these events effectively abrogates the migration of the receptor to the cell membrane and the stimulation of the membrane specific signaling pathway (129). The importance of striatin in mediating ERs correct localization at plasma membrane was also demonstrated since disruption of ER-striatin interactions abrogated E2-mediated protection against vascular injury (101). More recently, the central role of estrogen-mediated plasma membrane signaling in EC proliferation and migration was further demonstrated by the generation of a mutant version of ERα (KRR ERα), specifically defective in this rapid signaling pathway (103).

**Table 1:** Roles played by estrogen receptors in cardiac function in response to hormonal stimuli: studies in genetically modified animals.

**Mouse model**	**Genetic feature**	**Vascular phenotype and estrogen response**
ERα-Neo-KO	Insertion of neomycin resistance cassette into ESR1 exon 1 resulting in an ERα mutant form lacking the functional AF-1 (78).	Protection of carotid arterial from injury (79). Preserved endothelial NO production (80). Heart functional recovery after I/R in ERαKO female hearts similar to that in WT (75). More severe cardiac damage following I/R injury in male mice (73). Cardiac growth unresponsive to E2 treatment (74).
ERα ^−/−^	Insertion of neomycin resistance cassette into ESR1 exon 2 resulting in complete deletion of ERα (81).	Loss of re-endothelialization process (82). Inhibition of NO production in aorta (83). No protection in vascular injury (84). Reduced coronary capillary density associated to decreased VEGF expression and signaling (85).
ER1KO	Targeted mutation of ERα (71)	Decreased heart functional recovery in female ER1KO in comparison to female WT (71).
NERKI ^+/−^ or ERα^−/A^ (KI)	Mutated allele in DBD (E207A/G208A, or AA) introduced onto the ERα^−/−^ background (86, 87).	Not determined
ENERKI	Mutation in LBD domain of ERα (G525L) (88).	Not determined
KIKO	Generated by crossing NERKI^+/−^ with ERα ^+/−^ mouse model (89).	Not determined
ERα(^EAAE/EAAE^) transgenic (KI)	Mutation of four amino acid in the DNA recognition helix (Y201E, K210A, K214A, R215E) (90, 91).	Not determined
H2NES ERα mutant	Insertion of some point mutations in the NLS combined with a nuclear export signal (NES) in the D-domain (92, 93).	Not determined
ERαAF-1^0^	Deletion of AF1 domain (amino acids 2-148) (94).	Preserved endothelial NO production and re-endothelialization process and prevention of atheroma (94). Inhibition of neointimal hyperplasia protection in VSMC ERα AF-1 (95).
RαAF-2^0^	Deletion of AF2 domain (aa 543–549) (96).	Preserved endothelial repair but failed atheroprotective action (96). Unresponsive to estrogens for beneficial arteriolar effects (97).
MOER	Expression of the ERα E domain (LBD-AF2) containing multiple palmitoylation sites in an ERα^−/−^ background (98).	Not determined
NOER or C451A-ERα	Mutation of palmitoylation site of ERα.	Absence of eNOS phosphorylation, vasorelaxation, acceleration of endothelial healing (99, 100). Fully responsive to prevent atheroma and Ang II–induced hypertension (97).
DPM	Overexpression of the Disrupting Peptide Mouse (DPM) (aa 176–253) to inhibit ERα interaction with striatin (101).	Inability to stimulate EC migration and to inhibit VSMC growth *in vitro*. Loss of protection against vascular injury *in vivo* (101).
(KRR^ki/ki^)	Mutated ERα (KRR) introduced onto the ERα^−/−^ background under the control of the endogenous ERα promoter (102).	Not determined (102). EC (KRR ERα) inability to proliferate and migrate (103).
csERα-OE	Conditioned cardiomyocyte-specific overexpressing ERα (csERα-OE).	Increased LV mass, LV volume and cardiomyocytes length in both sexes. Attenuated fibrosis and increased angiogenesis and lymphangiogenesis in female ERα-OE after MI (104).
csERα^−/−^	Cardiomyocyte-specific ERαKO (csERα^−/−^).	Sex-differences in multiple structural parameters of the heart, with minimal functional differences. Identification of different gene networks potentially involved in cardiac biology (105).
ERβKO	Insertion of neomycin resistance cassette into exon 3 of ESR2 (81, 106). Expression of several transcript variants lacking exon 3.	Conserved inhibition of VSMC proliferation and increase in vascular medial area (106, 107). Vasoconstriction and VSMC abnormalities (106, 108). Defects in heart morphology and increased hypertension with aging (106, 109). More severe heart failure with increased mortality after MI in female KO mice (106, 110). Less heart functional recovery after I/R in ERβKO female hearts compared to WT (75, 106). Loss of inhibition of Ang II-induced hypertrophy (106, 111). Conserved accelerated re-endothelialization in female mice (81, 82). Absence of atherosclerosis protection (112).
ERβKO	Deletion of exon 3 by Cre/LoxP-mediated excision (113, 114). Residual deleted ERβ protein in the prostate tissue (114).	No abnormalities of heart morphology, morphometry, and ultrastructure in 16-month-old males (113). No vascular phenotype determined (114). No cardioprotective effects of E2 on LV hypertrophy (115).
csERβ-OE	Conditioned cardiomyocyte-specific overexpressing ERβ (csERβ-OE).	No differences in heart structure and function compared with WT mice. Improved survival and cardiac function in both sexes compared to the WT counterparts after MI. Attenuated cardiac fibrosis in males csERβ-OE mice (116).
GPER KO1-4	Deletion of GPER30 open reading frame to generate KO1 (117) KO2 (118) and KO3 (119). Insertion of full-length lacZ transcript insertion, retaining the C-term portion of the protein in KO4 (120).	Absence of beneficial effects on vascular tone and blood pressure (117, 121). Increased atherosclerosis progression (117, 122). Abrogated vasodilator response (117, 123). Increased blood pressure and vascular resistance with aging (118). Loss of cardioprotection against I/R injury in male mice (118, 124). Impaired LV cardiac function in male KO mice (119, 125). No evident blood pressure problems in younger mice (120).
csGPER-KO	Cardiomyocyte-specific GPER KO.	Alterations of cardiac structure and functional impairment. LV dimension more affected in male KO mice compared to female ones. Differential gene expression profiles affecting multiple transcriptional sex-related networks (126).

Furthermore, some mouse models have been created in order to dissect the different pathways triggered by the nuclear and the non-nuclear ER signaling. The MOER mouse model (98), expressing only the membrane domain (LDB-AF2 domain), showed a phenotype that was very similar to that of ERα^−/−^. However, these mice were still able to regulate some metabolic pathways in response to estrogen treatment (130). On the other hand, in murine models expressing only ERα nuclear mutant (e.g., NOER) the beneficial vascular effects of estrogen were lost (99). More recently, further studies on a different ERα knockout (KO) mouse model have allowed to better define the role played by the nuclear (ERαC451A) and non-nuclear (ERαAF2°) estrogen signaling in arterial protection (97).

ERβ KO mouse models have also been proposed in order to better define the metabolic and vascular activity of ERβ receptors (81, 106). Although showing a less severe phenotype compared with ERα KO, these mice were characterized by abnormalities of heart morphology (109), increased severity of heart failure (HF) after MI as well as less functional recovery after I/R, especially in female mice (75, 110). However, other studies failed to reveal a specific protective role of ERβ in atherosclerosis (112) or in vascular injury (107). Since these two murine models displayed alternative splicing transcripts, additional KO models were also generated (113, 114). Indeed, in these mice the expression of a portion of ERβ in the prostate was observed, suggesting the presence of some still active minor transcripts (114). However, despite being sterile, these KO mice showed a correct development of the main organs and a normal homeostasis of the different body systems. In particular, Antal and coworkers reported the absence of heart abnormalities in 16-month-old male mice (113).

As regards GPER KO, four different mouse models were generated. However, no evident phenotype changes in terms of viability or reproductive function were observed. Three of them (117–119) did not express GPER, whereas the fourth mouse model synthesized a lacZ reporter fused with the C-terminal portion of GPER, leaving open the question whether this truncated protein could play a functional role (120). Several vascular problems, in terms of increased blood pressure and atherosclerosis, were shown in the first two models of GPER KO (117, 118, 121–124).

In order to avoid systemic influence on ERs protective effects on the heart, different mouse models were generated characterized by genetically modified cardiomyocytes. Therefore, CMs overexpressing ERα (104) or with defective expression of ERα (105) were established. They demonstrated an important role of ERα in cardiac mass development in both sexes. In particular, ERα gain of function showed a more efficient cardiac repair in female mice in comparison with male mice after ischemic injury (104). As concerns ERβ, mice overexpressing this receptor in the cardiomyocytes (116) showed an improved survival after a MI in both sexes, compared with the wild type counterparts. In addition, a more recent mouse model carrying cardiomyocyte-specific GPER-KO showed structural and functional cardiac alterations in both sexes with LV defect more pronounced in the male mice characterized by an inadequate heart remodeling (126). As extensively discussed in a very recent review (131), ER cardioprotective potential should be investigated in more detail in order to more precisely define the role played by each receptor in the heart integrity and function.

### Estrogen Regulatory Role on the Heart

Cardiovascular repair and regeneration is reached by a series of mechanisms that include, on one hand, the reduction of inflammation and the formation of new vessels, on the other the survival and protection of cardiomyocytes (CMs), the activation of a cardiomyogenic process and a sort of cellular anti-aging program, i.e., an antioxidant activity. In this regard, E2 exerts many pleiotropic effects, some of which have a beneficial role on vascular endothelial cells as well as on smooth muscle and cardiac cells.

The role played by estrogens in cardioprotection against I/R injury pass through nitric oxide (NO) production (132). NO seems to play several potential beneficial roles in the cardiovascular system. Estrogen increases NO bioavailability in the vascular system through both the signaling pathways (genomic and non-genomic). Through the non-genomic signaling, E2 binding to ERα lead to endothelial nitric oxide synthase (eNOS) phosphorylation and activation. Upon estrogen binding, caveolae membrane-associated ERα activates Src family tyrosine kinases, PI3K/AKT kinase, and ERK1,2 to stimulate eNOS in NO production (133, 134). In line with these *in vitro* studies, an increase of eNOS activity together with a decreased number of leukocytes normally accumulating on the vascular wall after I/R injury has been observed in mice treated with estrogen. Accordingly, treatment with inhibitors of PI3K or eNOS abolished estrogen vascular protective effect (135). It has also been reported that, in human EC, calcium ions, of great importance in the regulation of nitric oxide synthase activity, increase rapidly at physiological estrogen concentrations (136). This modulation of Ca^2+^ homeostasis is ERα-dependent as demonstrated by using ERα KO cells (137). More recently, an estrogen-dendrimer conjugate (EDC) was reported to selectively activate extra-nuclear ER, in both EC and CMs. However, it seems able to attenuate infarct size in mice lacking ERα expression in CMs but not in mice lacking ERα expression in EC (138). This suggests that a different mechanism may be responsible for cardioprotection in CMs and EC.

As far as VSMC was concerned, it was observed that their proliferation was strictly controlled by kinase-mediated signal transduction. This kinase activity was in turn regulated by a balance between phosphorylation and dephosphorylation events. Indeed, the estrogen-mediated phosphatase activation determines the inhibition of several kinases leading to cell proliferation and migration block. In particular, VSMC proliferation was inhibited by phosphatase 2A, whose activation was mediated by interaction with ERα (139). More recently, in a mouse model with the selective blockade of the membrane-initiated ER signaling (KRR^ki/ki^) the central action of PP2A in metabolic homeostasis has been reported (102).

Non-genomic signaling pathways seem to have a key role in mediating the regulatory action of estrogens in all the cellular components of the cardiovascular system. As a matter of fact, the blockade of the non-genomic signaling impaired the transcriptional response of genes involved in the vascular function, indicating that the rapid estrogen signaling may contribute to physiological vascular gene regulatory activity (101, 103). Nonetheless, a strong cross talk between the genomic and non-genomic estrogen pathways has been hypothesized.

As concerns GPER, its vasodilatory effect was analyzed by using GPER agonists *in vitro* (140) or in KO mouse models, as discussed above (117, 118, 121). Furthermore, accumulating literature indicates that GPER vasorelaxation *in vivo* could be mediated by both endothelium-dependent and endothelium-independent mechanisms. In the former case, as in the arteries' relaxation, estrogen binds to GPER and leads to the production of nitric oxide in coronary EC by eNOS activation (140). In the endothelium-independent way, the E2-GPER effect on smooth muscle cells relaxation is mediated by the stimulatory activity of calcium- and voltage-activated potassium channels (67). The observed antiproliferative effect of GPER on EC (141) may provide an optimal balance for the opposite effects exerted by ERs on these cells. For example, in rat aortic EC, E2 elicits opposite effects depending on whether the signal depends on ERα or GPER (142). In fact, as for VSMC, GPER seems to act in concert with ERs in inhibiting proliferation and stimulating the differentiation rate of these cells (121, 143, 144). A GPER-mediated paradoxical effect of estrogen in vascular function (relaxation vs. contraction) was also described in porcine coronary arteries, involving the signaling pathway that passes through the transactivation of EGFR (145).

Several studies have shown that estrogen prevents cardiac hypertrophy, in particular through ERβ signaling (146). Firstly, it has been shown how ERs stimulate the production of the myocyte-enriched calcineurin-interacting protein (MCIP1), an inhibitor of calcineurin activity via PI3K. In this way, ERβ signaling blocks the angiotensin II (Ang II)- or endothelin-1 (ET-1)- mediated stimulation of key hypertrophy and ventricular remodeling genes in CMs (146). Thereafter, E2 inability to prevent Ang II-induced hypertrophy and fibrosis in ERβ KO mice was also demonstrated, underscoring the relevance of ERβ in counteracting cardiac hypertrophy (111, 147). Accordingly, the same authors demonstrated that E2 exerted regulatory effects on the synthesis, localization and function of histone deacetylase (HDA) class I (pro-hypertrophic) and class II (anti-hypertrophic), important modulators of cardiac hypertrophy. In this context, ERβ activation suppressed Ang II-induced HDAC2 (class I) production and de-repressed the opposite effects of Ang II on HDAC4 and HDAC5 (class II) (148). The key role of ERβ on hypertrophy was confirmed *in vivo* in hearts derived from ERβ KO mice (111, 147, 148). It is well-known that Ang II stimulates cardiac hypertrophy, in part by inhibiting KLF15 expression. In turn, E2 binding to ERβ appears able to reverse Ang II action, allowing KLF15 transcriptional regulation activity on cardiac hypertrophic gene expression (149). Furthermore, ERβ plays an anti-fibrotic role influencing cardiac fibroblast homeostasis down-modulating TGFβ expression and signaling, otherwise stimulated by Ang-II (150). As regards cardiac fibroblasts, it has very recently been hypothesized that E2, either via ERα or ERβ signaling, could exert opposite effects on the synthesis and secretion of key components of the extracellular matrix, i.e., collagen I and III, by these cells (151). Regarding the debated question dealing with the possibility that GPER could or not activate an autonomous signal, it has been observed that estradiol treatment of infarcted rats improved ventricular remodeling triggering both GPER and ERα activity. Indeed, both receptors activate their membrane-specific signaling that converged into the common PI3K/AKT/eNOS pathway (152). As regards CMs, GPER was suggested to activate signaling of PI3-kinase contributing to cardioprotection in females (153). Interestingly, the PI3K pathway seems to be strictly related to autophagic processes involved in cardioprotection (154), and it has been very recently reported that GPER could counteract CM hypertrophy by up-regulating the PI3K-AKT-mTOR signaling pathway, therefore modulating autophagy (155). A further mechanism of estrogen-induced cardioprotection involving GPER was investigated using its agonist (called G1) in a mouse model of I/R injury. Both G1 and E2 exerted a cardioprotective activity by inhibiting mitochondria permeability transition pore opening that normally leads to apoptotic cell death of CMs after I/R injury (47, 156). A further study demonstrated that post-ischemic GPER activation, preserving mitochondrial structural integrity, decreased ROS production and mitophagy, resulting in reduced myocardial infarct size in both sexes (157). As discussed before, specific GPER KO cardiomyocytes exhibited left ventricular dysfunction and adverse remodeling more pronounced in male KO mice than in female. Furthermore, DNA microarray analysis revealed gene expression differences between sexes, with particular reference to the mitochondrial and inflammatory pathways (126). Finally, the pivotal role of GPER and the involvement of Notch1 pathway in mediating physiopathology of female rat hearts were hypothesized (158).

The effects of E2 on myocyte regeneration have also been investigated. Several studies focused on cardiomyogenesis have established that the genesis of new cardiomyocytes from the preexisting cardiomyocyte pool occurs at a low rate (159, 160). The presence of multipotent cardiac stem cells (CSCs), normally residing within the cardiac niche, has extensively been studied (161, 162) as well as CSC induction to proliferate, migrate, and undergo lineage commitment in response to infarction injury (163). Accordingly, it has been demonstrated that CSCs isolated from adult rodent hearts express stem cell surface markers (c-Kit/Sca-1) and display several stem cell functions (161, 164, 165). Indeed, c-Kit+ precursor cells, which accumulate in the infarcted area, showed increased ERα expression, suggesting a direct effect of E2 on cardiac progenitor cells *in situ* (166).

Estrogen-replacement therapy and acute myocardial infarction were evaluated in a rat experimental model. It has been observed that estrogen-replacement therapy increases the homing of bone marrow stem cells into myocardium and stimulate angiogenesis enhancing ERα and ERβ expression (167). The possibility of ERα-mediated paracrine cardioprotective function has been proposed as one of the major mechanism used by post-infarct cardiac c-kit+ cells (i.e., inducing CM survival). Accordingly, infusion of E2 treated-CSCs into the isolated mouse hearts after acute I/R gave rises to a powerful protective effect probably due to a major production of CSC-derived protective factors (168).

## Mitochondria as Subcellular Targets of Estrogen

Mitochondria drive different cellular processes by providing chemical energy and they are particularly important in heart muscle cells where mitochondrial dysfunction is associated with important pathological changes leading to impaired cardiac function (169). In fact, dysfunctional mitochondria would ultimately lead to myocardial cell apoptosis and death during I/R injuries.

On the other hand, autophagy, characterized by the formation of autophagosomal vesicles containing degenerating cytoplasmic contents, is considered primarily as a cytoprotective process. Particularly, mitophagy, a selective form of autophagy, represents a protective mechanism that contributes to eliminate damaged mitochondria thus reducing mitochondria-mediated apoptosis and necrosis in the myocardium (170). Accordingly, it has been suggested that autophagy counteracts mitochondrial dysfunction by autophagosome formation, possible embedding of damaged mitochondria in autophagolysosomes and their digestion. This allows the cells to remove injured mitochondria that often represent a source of ROS. During I/R, mitochondria suffer a deficiency to supply the CMs with chemical energy also contributing to oxidative stress and to the cytosolic ionic alterations, especially of Ca^2+^ (171). Interestingly, it has been hypothesized that different types of cardiomyocyte calcium channels could exhibit a marked sexual dimorphism and that their function could be regulated by ERα, ERβ, and GPER, i.e., by non-nuclear estrogen receptor signals (131).

Sex plays a pivotal role in the cardiac tolerance to I/R injury, and it has been reported that male myocardium is more sensitive than the female one. Recent studies have suggested that mitochondria are a major target of cardioprotective signaling (31, 172). Furthermore, numerous studies have suggested that in females mitochondria could be modified and less sensitive to I/R injury. In addition, it was reported that mitochondria from females undergo several posttranslational modifications of enzymes involved in the redox metabolism generating less ROS during the reoxygenation phase following ischemia (173–176).

In particular, Colom and co-authors (174) demonstrated a significant sex difference in the function of cardiac mitochondria. Female rats showed minor cardiac mitochondria content and produced less H_2_O_2_ than male rats. On the other hand, male myocytes, thanks to the higher density of β-adrenergic receptors, are more responsive to β-adrenergic stimulation than females. This induces an increase in the influx of Ca^2+^ in cardiac cells. Male myocytes are thus particularly prone to calcium overload (177). According with this, it was observed an improved survival of CMs overexpressing ERβ isolated from mice of both sexes, together with a significant reduction of the maladaptive remodeling and the recovery of cardiac function after MI in comparison with wild type CMs. These effects seem to be associated to a better maintenance of Ca^2+^ homeostasis and to less cardiac fibrosis following MI (116).

Mitochondria isolated from hearts of adult male and female rats differ in the sensitivity of the permeability transition pore (MPTP) to the calcium load. In particular, mitochondria isolated from female animals appear more resistant to swelling induced by high Ca^2+^ concentration. It can be hypothesized that the higher ischemic tolerance of female myocardium may be related to the lower sensitivity of MPTP to the calcium induced swelling. Accordingly, it has been observed that a specific ERβ agonist reduced mitochondria-mediated apoptosis and contribute to the preservation of mitochondrial integrity after I/R injury (178).

Bcl2 protein, located at mitochondrial membranes, provides protection against pro-apoptotic stimuli (179), and its expression level is associated with improved recovery of cardiac function after I/R, and reduced infarction area due to a reduced apoptotic cell death (180). Moreover, Bcl2 prevents permeabilization of the outer mitochondrial membrane (181) after I/R thus preventing the release of cytochrome c from mitochondria and subsequent apoptosis. To note, the expression of Bcl2 was found controlled by ERβ (182). It was also reported that the cardioprotection observed in female sex may be related to a greater protein expression of the sarcolemmal and mitochondrial K(ATP) channels. According with this, the blockade of K(ATP) channels significantly increased the damage in the female heart after I/R (183, 184).

Mitochondrial dynamics (i.e., fission/fusion processes) is critical for a correct mitochondrial function, and alterations of mitochondrial dynamics have been associated with neuropathies, non-alcoholic fatty liver disease progression, type 2 diabetes, and CVD (185–187). Very recently, an uncontrolled balance of mitochondrial dynamics was shown to contribute to cardiac dysfunction during I/R injury (188). Several proteins are involved in mitochondrial dynamics: for instance, mitofusins (MFN) and optic atrophy protein 1 (OPA1) participate to mitochondrial fusion process, while mitochondrial fission is manly orchestrated by dynamin-related protein 1 (DRP1) and fission protein 1 (FIS1) (185). Alterations in this mitochondrial dynamics produce altered mitochondria in their shape and size: a prevalence of fusogenic mechanisms favors the formation of a large mitochondrial network; on the contrary, if fission mechanisms prevail a mitochondrial fragmentation occurs (189). In different *in vivo* and *in vitro* models of ischemia or I/R, it was observed that the inhibition of DRP1 selectively blocks DRP1-dependent mitophagy, which is triggered to eliminate mitochondria damaged during the early phase of ischemia in the brain (190). After the inhibition of DRP1, CMs were found to show a significant decrease of oxygen consumption with a negligible alteration of ATP production after I/R (191). Accordingly, in I/R-induced alterations of CMs, mdivi-1, a chemical inhibitor of the mitochondrial fission protein DRP1 that induces mitochondria elongation (192), preserved the mitochondrial structure and significantly reduced the myocardial infarction area (193, 194). Current studies thus indicate that several chemical compounds prevent the alterations of mitochondrial dynamics. However, further toxicological and pharmacokinetic studies are needed before their clinical use.

A very interesting role of sex hormones was reported in mitochondrial biogenesis occurring in the right ventricle after the heart failure associated with pulmonary hypertension. In particular, Liu and co-workers, by studying ovariectomized female rats, found that estrogen therapy counteracted the loss mitochondrial mass and maintain the cardiac oxidative metabolism. They therefore hypothesized that estrogen could prevent maladaptive remodeling of the right ventricle that often lead to the severe dysfunction frequently associated with pulmonary hypertension (195). Furthermore, it has also been suggested that E2 induces mitochondriogenesis in H9c2 cultured cardiomyocytes through the increase of PGC-1α expression. This effect seems to be mediated by GPER, since specific agonists of this receptor mimic the activity of estrogen (196). However, it should be underlined that other authors (197), in a study in a murine model of hemorrhagic trauma, reported that the effect exerted by estrogen on mitochondrial biogenesis and function at the cardiac level is mediated by both ERα and ERβ.

**Figure 2:**
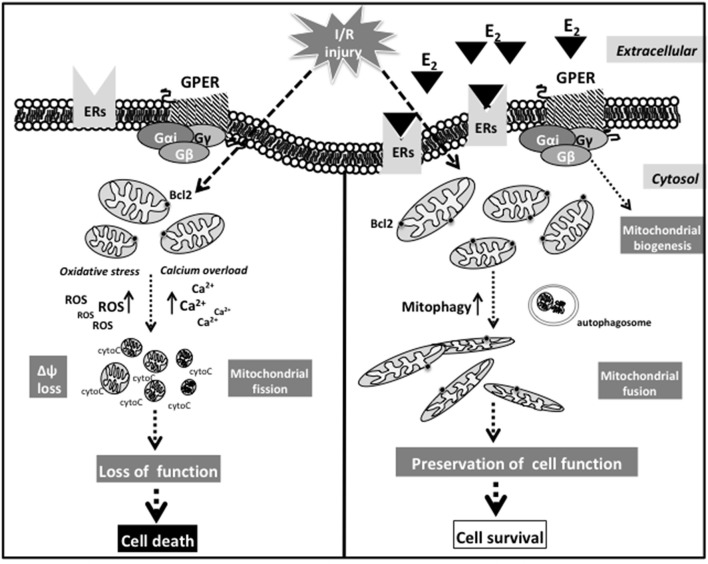
Schematic picture suggesting the possible sequence of events at mitochondrial level after I/R injury in response to activation of the putative non-nuclear ER pathway.

Although a direct or indirect influence of estrogens on mitochondrial dynamics has not yet been observed in cardiac models, it was found that I/R injury increased ROS production, mitochondrial fission, and increased levels of DRP1 in cardiomyocytes (198). Moreover, in a DRP1 KO mouse model, a cardiac-specific impairment of left ventricular functions has also been observed. These mice died within 13 weeks through the suppression of autophagic flux, thus underlining the pivotal role of autophagy or mitophagy in CM homeostasis (e.g., maintaining the ionic equilibrium) (199). A schematic picture suggesting the possible sequence of events at mitochondrial level after I/R injury in response to activation of the putative non-nuclear ER pathway is reported in Figure 2.

## Estrogen Regulated miRNAs and Their Effect on Myocardium and Cardiac Vascular System

Estrogens/estrogen receptor interaction regulates cardiovascular function through either gene expression or epigenetic mechanisms. This last mechanism of action is also dependent on miRNA action (200). miRNAs are highly conserved short non-coding RNAs (19-25 nucleotides) that control many developmental and cellular processes in eukaryotic organisms by post-transcriptional regulation of mRNAs by binding to their 3' untranslated regions, thus triggering their translational inhibition with or without RNA degradation. miRNA expression is strongly regulated at different levels, e.g., during development and for tissue specific functions (201).

Of relevance is, in fact, the role of miRNAs in regulating vascular cell aging, which in women after menopause appears similar to that detected in men (202). This obviously supports the knowledge of a regulatory role of estrogen in fertile woman. Notably, this different regulation might also rely on sex-linked miRNAs. Actually, about 120 miRNAs have been identified on human X chromosome, whereas only 4 on the Y chromosome. This appears as an intriguing result *per se*. In addition, although random X-inactivation should equilibrate female and male expression levels, a number of unbalancing mechanisms have emerged so far. In fact, genes escaping X chromosome inactivation could play a critical a role. Moreover, the number of these genes increases with age and it has been suggested that this could lead to an increased susceptibility of women to inflammatory and autoimmune disease (203). In this field, the interesting study of Florijn and co-workers remarked the harmful effects of the X-linked miRNAs in cardiovascular disease suggesting that the sex-biased miRNA network could play a key role in heart failure with preserved ejection fraction observed in women (202). This hypothesis is only partially in accord with the suggested protective effects of estrogen regulated miRNAs reported elsewhere. Furthermore, estrogens modulate miRNA profiles also during their maturation pathway (204). Numerous lines of evidence underline the importance of estrogen therapy in postmenopausal women to restore the correct level of miRNA expression among many other aging-related physio-pathological aspects. Estrogen protective action on cardiac vascular system has prevalently been associated with the ERα signaling that is responsible of vasodilation, inhibition of inflammation and regulation of the oxidative stress also blocking apoptosis. All these processes play a role in preserving the correct function of endothelial cells, modulating vasoconstriction and inhibiting proliferation of VSMC. Specific miRNA signatures have been associated with cardiac and vascular aging under estrogen control (202). Some estrogen regulated miRNAs and their effect on cardiac and vascular cells are reported in Table 2.

**Table 2:** Some estrogen regulated miRNAs and their effect on myocardium and cardiac vascular system cells.

**miRNA**	**Vascular cells**	**Functions**	**Targets**	**References**
miR-126-3p	Endothelial cells	Migration Angiogenesis	Spred1	(205)
miR-221&-222	Endothelial cells	Inflammation	ETS-1	(206)
miR-106b	Endothelial cells VSMC	Apoptosis	PTEN	(207)
miR-143/-145	VSMC	Proliferation Contraction	ACE	(208)
miR-30	Endothelial cells VSMC	Angiogenesis Apoptosis Inflammation	Ang2	(209)
miR-203	VSMC	Proliferation	SRC, ERK	(210)
miR-144	Endothelial cells VSMC	Inflammation Metabolism	COX2 Rac1 ABCA1	(211) (212) (213)
miR-146a	Endothelial cells	Inflammation Senescence	TRAF6, IRAK1 NOX4	(214) (215)
miR-21	VSMC Fibroblast	Inflammation Proliferation	PPARα, Spry1 NF1B,CDC25A	(216) (217) (218)
miR-125	Endothelial cells	Angiogenesis	RTEF-1, VEGF	(219)
miR-34	Endothelial cells	Senescence Inflammation	SIRT-1	(220, 221)

MI is consequent to a protracted ischemic injury of vasculature and hypoxic conditions that are characterized by continuous deficit of cardiomyocyte oxygenation and inflammation in the infarcted area. This picture is amplified by increased oxidative stress, i.e., ROS production, and cardiac muscle cell death (222). In the attempt to reduce tissue damage, the infarcted heart undergoes a cardiac self-remodeling that frequently results in increased fibrosis, dilated cardiomyopathy and heart failure (HF) (223). In this context, miRNA roles have broadly been investigated using both cardiac cell cultures and mouse models of cardiac infarction. It is now clear that miRNAs are implicated in cardiac proper functions as well as in pathogenesis of cardiac cell injury, leading to HF. For instance, some miRNAs have directly been associated with estrogen cardioprotective action against oxidative stress. The cystathionine-γ-lyase (CSE), the enzyme involved in cardioprotective H2S generation (224), is indirectly regulated by miR-22 levels as miR-22 specifically down-regulates the Sp1 transcription factor, involved in CSE transcription. Indeed, 17β-estradiol treatment determines down-modulation of this miRNA by ERα action, thus reconstituting Sp1 levels both in cultured cardiomyocytes and in ovariectomized rat hearts (225).

An independent risk factor for HF, and consequently for cardiovascular morbidity and mortality, is cardiac hypertrophy, either concentric or eccentric. The former consists of an increase in ventricular wall thickness without chamber enlargement. The latter promotes chamber dilation with no increase or even decrease of left ventricular wall thickness (226). This remodeling is characterized by age-specific relative changes in LV mass, volumes, and chamber performance during diastolic and systolic function. Differences of this remodeling between pre- and post-menopausal women suggest a key role for estrogen. Indeed, E2 deficiency in the heart of ovariectomized mice increases the age-related ventricular concentric remodeling that, at sub-cellular level, is underlined by the functional impairment of mitochondria.

The molecular mechanisms associated with this ventricular dysfunction have also been correlated to miR-23a levels. In absence of estrogens, miR-23a high level in cardiomyocytes directly targets peroxisome proliferator-activated receptor-γ co-activator 1-α (PGC-1α) down-regulating its expression. This protein is a modulator of mitochondrial function and its heart-specific deletion has recently been associated to cardiac dilation with LV thinning (227). However, as indicated by Sun and colleagues, the E2 deficiency might mediate a possible role of PGC-1α also in concentric remodeling through the miR-23a dependent reduction (228).

A rat model of myocardial ischemia showed that mortality was increased when accompanied with estrogen deprivation (due to ovariectomy). This elevated mortality was associated with miR-151-5p down-regulation. This miRNA binds to the 3′UTR of FXYD1, the gene codifying for phospholemman protein (PLM, an important regulator of ion transport and a substrate for protein kinases A and C), inhibiting its expression. PLM is known to alter cardiac membrane excitability. Thus, in the ovariectomized myocardial ischemic group of animals, the absence of estrogen, reducing miR-151-5p levels favored PML increase, with Ca^2+^ accumulation in cardiomyocytes eventually exacerbating cardiac malfunction (229).

Finally, a recent miRNA specific microarray study on cardiomyocytes treated or not with estrogen showed an increased expression of a further miR: the miR-494. The authors correlated the expression of this miRNA with estrogen dependent cardioprotection and identified in the nuclear factor kappa B (NF-κB) repressing factor (NKRF) the specific target of this miR in cardiomyocytes. In brief, miR-494 overexpression could mimic the estrogen specific cardioprotection reducing the oxidative stress-induced injury (230).

## Conclusions

In this paper we summarized some molecular mechanisms that lead to favorable or unfavorable evolution of remodeling of the heart after injury, e.g., in I/R, and how these mechanisms may depend on the effect of sex hormones, of estrogen non-genomic effects in particular. On the basis of the results described above, it appears well-documented that all cell components of the cardiovascular system (such as cardiomyocytes and fibroblasts, as well as endothelial and vascular smooth muscle cells) of males and females, also in virtue of their hormonal differences, differently counteract exogenous or endogenous insults. In this context, the emerging role of non-genomic effects of estrogen on cardiovascular cell homeostasis and remodeling could represent a formidable, novel challenge for this field of investigation. The idea that a prompt, very rapid, i.e., in seconds, response could be played out in order to counteract an injury appears fascinating as well as conceivable: the “classical” genomic activity of hormones appears, in our mind, as too slow in order to face damage and to survive. However, apart from the possible role of this mechanism in the evolution of the species (which should merit a specific discussion), the influence of the estrogen hormone and its pathways in determining cardiovascular cell homeostasis appears as pivotal and should merit more targeted analyses.

A last point deals with sex-specific studies. Although many experimental studies dealing with the analysis of sex differences in the cardiovascular system, either in physiological or in pathological conditions, have been published in the recent years, the molecular mechanisms whereby sex specificities may influence the remodeling and the adaptive response to injury are still to be defined in detail. As a general rule, these studies suggested resilience as a milestone of the female sex, including cellular and tissue responses to environmental insults. Experimental studies, e.g., in freshly isolated cells from males and females are, however, quite complicated. The use of “typical” cultured cells is in fact useless in this field since the great majority of cell lines derive from cancer cells or from established highly proliferating cell lines and we know that these models do not adequately apply to the study of vascular or cardiac cells. Hence, the main bias in the study of the different response of XX and XY cells is the availability of strong and effective cell models. Thus, the influence of hormones, sex hormones in particular, on cardiovascular cell system homeostasis in males and females represents a complex challenge that should properly be investigated in the next years by using cell pathology approaches in parallel with *in vivo* analyses. One further important issue should be referred to hormone variations in the lifespan of men and women that, due to its peculiarities, can be fully investigated neither *in vitro* nor *in vivo*. Consequently, translation of the results obtained in these experimental studies into clinical practice cannot be performed or it should be performed, when appropriate, very carefully. Notwithstanding this, experimental studies appear indispensable: clinical data are often descriptive rising questions to which mechanistic studies could try to answer. To do this, preclinical studies that incorporate both sexes will be crucial to allow the translation of information from basic research to clinical practice.

## Author Contributions

RP expert in the field of experimental model studies, i.e., animal studies. GMat looked at the aspects referred as to the role of micro RNA. PM followed the aspects dealing with the role of mitochondria in cellular remodeling. GMar contributed for the clinical aspects of cardiovascular disease. WM and AC conceived the work and supervised the manuscript.

### Conflict of Interest

The authors declare that the research was conducted in the absence of any commercial or financial relationships that could be construed as a potential conflict of interest.

## Abbreviations

Ang II: angiotensin II
CF: cardiac fibroblasts
CMs: cardiomyocytes
CVD: cardiovascular diseases
CSE: Cystathionine-γ-lyase
DRP1: dynamin-related protein 1
EC: endothelial cells
eNOS: endothelial nitric oxide synthase
ET-1: endothelin-1
E1: estrogen
E2: 17β-estradiol
E3: estriol
ER: estrogen receptor
GPER: G protein-coupling estrogen receptor
FIS1: fission protein 1
HF: heart failure
IL: interleukin
I/R: ischemia/reperfusion
KO: knockout
LV: left ventricle
MI: myocardial infarction
MMPs: matrix metalloproteinases
MISS: membrane-initiated steroid signal
MPTP: mitochondria permeability transition pore
MFN: mitofusins
CSC: multipotent cardiac stem cells
NF-κB: nuclear factor kappa B
NO: Nitric oxide
OPA1: optic atrophy protein 1
PGC-1α: peroxisome proliferator-activated receptor-γ co-activator 1-α
ROS: reactive oxygen species
VSMC: vascular smooth muscle cells.

## References

[1] Townsend N Nichols M Scarborough P Rayner M Cardiovascular disease in Europe—epidemiological update. Eur Heart J. (2015) 36:2696–705. 10.1093/eurheartj/ehv428 26306399

[2] Garcıa M Mulvagh SL Merz NB Buring JE Manson JE. Cardiovascular disease in women. Clin Perspect Circ Res. (2016) 118:1273–93. 10.1161/CIRCRESAHA.116.307547 27081110 PMC4834856

[3] Regitz-Zagrosek V. Therapeutic implications of the gender-specific aspects of cardiovascular disease. Nat Rev Drug Discov. (2006) 5:425–38. 10.1038/nrd2032 16672926

[4] Mozaffarian D Benjamin EJ Go AS Arnett DK Blaha MJ Cushman M American Heart Association Statistics C and Stroke Statistics S, Heart disease and stroke statistics-−2015 update: a report from the American Heart Association. Circulation. (2015) 131:e29–322. 10.1161/CIR.0000000000000152 25520374

[5] Yeh RW Sidney S Chandra M Sorel M Selby JV Go AS. Population trends in the incidence and outcomes of acute myocardial infarction. N Engl J Med. (2010) 362:2155–65. 10.1056/NEJMoa0908610 20558366

[6] Tamargo J Rosano G Walther T Duarte J Niessner A Kaski JC. Gender differences in the effects of cardiovascular drugs. Eur Heart J Cardiovasc Pharmacother. (2017) 3:163–82. 10.1093/ehjcvp/pvw042 28329228

[7] Abajobir AA Abbafati C Abbas KM Abd-Allah F Abera SF Aboyans V Global, regional, and national age- sex specific mortality for 264 causes of death, 1980–2016: a systematic analysis for the global burden of disease study 2016. Lancet. (2017) 390:1151–210. 10.1016/S0140-6736(17)32152-9 28919116 PMC5605883

[8] Frangogiannis NG. The inflammatory response in myocardial injury, repair, and remodelling. Nat Rev Cardiol. (2014) 11:255–65. 10.1038/nrcardio.2014.28 24663091 PMC4407144

[9] Neri M Riezzo I Pascale N Pomara C Turillazzi E. Ischemia/Reperfusion Injury following acute myocardial infarction: a critical issue for clinicians and forensic pathologists. Mediators Inflamm. (2017) 2017:7018393. 10.1155/2017/7018393 28286377 PMC5327760

[10] Lindsey ML. Assigning matrix metalloproteinase roles in ischaemic cardiac remodelling. Nat Rev Cardiol. (2018) 15:471–79. 10.1038/s41569-018-0022-z 29752454 PMC6203614

[11] Voorhees AP DeLeon-Pennell KY Ma Y Halade GV Yabluchanskiy A Iyer RP. Building a better infarct: modulation of collagen cross-linking to increase infarct stiffness and reduce left ventricular dilation post-myocardial infarction. J Mol Cell Cardiol. (2015) 85:229–39. 10.1016/j.yjmcc.2015.06.006 26080361 PMC4530076

[12] Scotland RS Stables MJ Madalli S Watson P Gilroy DW. Sex differences in resident immune cell phenotype underlie more efficient acute inflammatory responses in female mice. Blood. (2011) 118:5918–27. 10.1182/blood-2011-03-340281 21911834 PMC5363818

[13] Rathod KS Kapil V Velmurugan S Khambata RS Siddique U Khan S. Accelerated resolution of inflammation underlies sex differences in inflammatory responses in humans. J Clin Invest. (2017) 127:169–82. 10.1172/JCI89429 27893465 PMC5199722

[14] Pessôa BS Slump DE Ibrahimi K Grefhorst A van Veghel R Garrelds IM. Angiotensin II type 2 receptor- and acetylcholine-mediated relaxation: essential contribution of female sex hormones and chromosomes. Hypertension. (2015) 66:396–402. 10.1161/HYPERTENSIONAHA.115.05303 26056343

[15] Straface E Gambardella L Pagano F Angelini F Ascione B Vona R. Sex differences of human cardiac progenitor cells in the biological response to TNF-α treatment. Stem Cells Int. (2017) 2017:4790563. 10.1155/2017/4790563 29104594 PMC5623773

[16] Matarrese P Colasanti T Ascione B Margutti P Franconi F Alessandri C. Gender disparity in susceptibility to oxidative stress and apoptosis induced by autoantibodies specific to RLIP76 in vascular cells. Antioxid Redox Signal. (2011) 15:2825–36. 10.1089/ars.2011.3942 21671802

[17] Malorni W Straface E Matarrese P Ascione B Coinu R Canu S. Redox state and gender differences in vascular smooth muscle cells. FEBS Lett. (2008) 582:635–42. 10.1016/j.febslet.2008.01.034 18242172

[18] Feng Y Bopassa JC. Oxygen surrounding the heart during ischemic conservation determines the myocardial injury during reperfusion. Am J Cardiovasc Dis. (2015) 5:127–39. 26309776 PMC4539099

[19] Levonen AL Hill BG Kansanen E Zhang J Darley-Usmar VM. Redox regulation of antioxidants, autophagy, and the response to stress: implications for electrophile therapeutics. Free Radic Biol Med. (2014) 71:196–207. 10.1016/j.freeradbiomed.2014.03.025 24681256 PMC4042208

[20] Corsetti G Yuan Z Romano C Chen-Scarabelli C Fanzani A Pasini E. Urocortin induces phosphorylation of distinct residues of signal transducer and activator of transcription 3 (STAT3) via different signaling pathways. Med Sci Monit Basic Res. (2019) 25:139–52. 10.12659/MSMBR.914611 31073117 PMC6532558

[21] Xiao J Ke ZP Shi Y Zeng Q Cao Z. The cardioprotective effect of thymoquinone on ischemia-reperfusion injury in isolated rat heart via regulation of apoptosis and autophagy. J Cell Biochem. (2018) 119:7212–17. 10.1002/jcb.26878 29932232

[22] Ye G Fu Q Jiang L Li Z. Vascular smooth muscle cells activate PI3K/Akt pathway to attenuate myocardial ischemia/reperfusion-induced apoptosis and autophagy by secreting bFGF. Biomed Pharmacother. (2018) 107:1779–85. 10.1016/j.biopha.2018.05.113 30257397

[23] Kubli DA Gustafsson AB. Cardiomyocyte health: adapting to metabolic changes through autophagy. Trends Endocrinol Metab. (2014) 25:156–64. 10.1016/j.tem.2013.11.004 24370004 PMC3951169

[24] Razzolini R Dal Lin C Gender differences in heart failure. Ital J Gender-Specific Med. (2015) 1:15–20. 10.1723/2012.21914

[25] Merlo M Nuzzi V Bessi R Fabris E Sinagra G Gender differences in heart failure. Ital J Gender-Specific Med. (2017) 3:141–3. 10.1723/2924.29392

[26] Gruber CJ Tschugguel W Schneeberger C Huber JC. Production and actions of estrogens. N Engl J Med. (2002) 346:340–52. 10.1056/NEJMra000471 11821512

[27] Nelson LR Bulun SE. Estrogen production and action. J Am Acad Dermatol. (2001) 45:S116–24. 10.1067/mjd.2001.117432 11511861

[28] Stocco C. Tissue physiology and pathology of aromatase. Steroids. (2012) 77:27–35. 10.1016/j.steroids.2011.10.013 22108547 PMC3286233

[29] Bell JR Mellor KM Wollermann AC Ip WT Reichelt ME Meachem SJ. Aromatase deficiency confers paradoxical postischemic cardioprotection. Endocrinology. (2011) 152:4937–47. 10.1210/en.2011-1212 22028441

[30] Jazbutyte V Stumpner J Redel A Lorenzen JM Roewer N Thum T. Aromatase inhibition attenuates desflurane-induced preconditioning against acute myocardial infarction in male mouse heart *in vivo*. PLoS ONE. (2012) 7:e42032. 10.1371/journal.pone.0042032 22876297 PMC3410886

[31] Schuit SC de Jong FH Stolk L Koek WN van Meurs JB Schoofs MW. Estrogen receptor alpha gene polymorphisms are associated with estradiol levels in postmenopausal women. Eur J Endocrinol. (2005) 153:327–34. 10.1530/eje.1.01973 16061840

[32] Rexrode KM Ridker PM Hegener HH Buring JE Manson JE Zee RY. Polymorphisms and haplotypes of the estrogen receptor-β gene (ESR2) and cardiovascular disease in men and women. Clin Chem. (2007) 53:1749–56. 10.1373/clinchem.2007.091454 17702854 PMC2085372

[33] Shearman AM Cooper JA Kotwinski PJ Miller GJ Humphries SE Ardlie KG. Estrogen receptor α gene variation is associated with risk of myocardial infarction in more than seven thousand men from five cohorts. Circ Res. (2006) 98:590–2. 10.1161/01.RES.0000210578.62102.a6 16484614

[34] Hammes SR Levin ER. Extranuclear steroid receptors: nature and actions. Endocr Rev. (2007) 28:726–41. 10.1210/er.2007-0022 17916740

[35] Pedram A Razandi M Deschenes RJ Levin ER. DHHC-7 and−21 are palmitoylacyltransferases for sex steroid receptors. Mol Biol Cell. (2012) 23:188–99. 10.1091/mbc.e11-07-0638 22031296 PMC3248897

[36] Barbati C Pierdominici M Gambardella L Malchiodi Albedi F Karas RH. Cell surface estrogen receptor alpha is upregulated during subchronic metabolic stress and inhibits neuronal cell degeneration. PLoS ONE. (2012) 7:e42339. 10.1371/journal.pone.0042339 22860116 PMC3409197

[37] Ortona E Gambardella L Barbati C Malorni W Membrane-associated functional estrogen receptors alpha are upregulated in cardiomyocytes underoxidative imbalance. IJC Metab Endocr. (2014) 5:67–9. 10.1016/j.ijcme.2014.08.008

[38] Maselli A Pierdominici M Vitale C Ortona E. Membrane lipid rafts and estrogenic signalling: a functional role in the modulation of cell homeostasis. Apoptosis. (2015) 20:671–8. 10.1007/s10495-015-1093-5 25637184

[39] Filardo EJ Thomas P. Minireview: G protein-coupled estrogen receptor-1, GPER-1: its mechanism of action and role in female reproductive cancer, renal and vascular physiology. Endocrinology. (2012) 153:2953–62. 10.1210/en.2012-1061 22495674 PMC3380306

[40] Revankar CM Cimino DF Sklar LA Arterburn JB Prossnitz ER. A transmembrane intracellular estrogen receptor mediates rapid cell signaling. Science. (2005) 307:1625–30. 10.1126/science.1106943 15705806

[41] Stice JP Knowlton AA. Estrogen, NFkappaB, and the heat shock response. Mol Med. (2008) 14:517–27. 10.2119/2008-00026.Stice 18431462 PMC2323333

[42] Safe S Kim K. Non-classical genomic estrogen receptor (ER)/specificity protein and ER/activating protein-1 signaling pathways. J Mol Endocrinol. (2008) 41:263–75. 10.1677/JME-08-0103 18772268 PMC2582054

[43] Acconcia F Kumar R. Signaling regulation of genomic and nongenomic functions of estrogen receptors. Cancer Lett. (2006) 238:1–14. 10.1016/j.canlet.2005.06.018 16084012

[44] Kumar P Wu Q Chambliss KL Yuhanna IS Mumby SM Mineo C Direct interactions with Gαi and Gβ*γ* mediate nongenomic signaling by estrogen receptor α. Mol Endocrinol. (2007) 21:1370–80. 10.1210/me.2006-0360 17405905

[45] Madak-Erdogan Z Kieser KJ Kim SH Komm B Katzenellenbogen JA Katzenellenbogen BS. Nuclear and extranuclear pathway inputs in the regulation of global gene expression by estrogen receptors. Mol Endocrinol. (2008) 22:2116–27. 10.1210/me.2008-0059 18617595 PMC2631368

[46] Prossnitz ER Barton M. Estrogen biology: new insights into GPER function and clinical opportunities. Mol Cell Endocrinol. (2014) 389:71–83. 10.1016/j.mce.2014.02.002 24530924 PMC4040308

[47] Bopassa JC Eghbali M Toro L Stefani E. A novel estrogen receptor GPER inhibits mitochondria permeability transition pore opening and protects the heart against ischemia-reperfusion injury. Am J Physiol Heart Circ Physiol. (2010) 298:H16–23. 10.1152/ajpheart.00588.2009 19880667 PMC2806134

[48] Prossnitz ER Maggiolini M. Mechanisms of estrogen signaling and gene expression via GPR30. Mol Cell Endocrinol. (2009) 308:32–8. 10.1016/j.mce.2009.03.026 19464786 PMC2847286

[49] Pupo M Vivacqua A Perrotta I Pisano A Aquila S Abonante S. The nuclear localization signal is required for nuclear GPER translocation and function in breast Cancer-Associated Fibroblasts (CAFs). Mol Cell Endocrinol. (2013) 376:23–32. 10.1016/j.mce.2013.05.023 23748028

[50] Grohé C Kahlert S Löbbert K Stimpel M Karas RH Vetter H. Cardiac myocytes and fibroblasts contain functional estrogen receptors. FEBS Lett. (1997) 416:107–12. 10.1016/S0014-5793(97)01179-4 9369244

[51] Taylor AH Al-Azzawi F. Immunolocalisation of oestrogen receptor beta in human tissues. J Mol Endocrinol. (2000) 24:145–55. 10.1677/jme.0.0240145 10657006

[52] Mahmoodzadeh S Eder S Nordmeyer J Ehler E Huber O Martus P. Estrogen receptor alpha up-regulation and redistribution in human heart failure. FASEB J. (2006) 20:926–34. 10.1096/fj.05-5148com 16675850

[53] Lizotte E Grandy SA Tremblay A Allen BG Fiset C. Expression, distribution and regulation of sex steroid hormone receptors in mouse heart. Cell Physiol Biochem. (2009) 23:75–86. 10.1159/000204096 19255502

[54] Yang SH Liu R Perez EJ Wen Y Stevens SM Jr Valencia T. Mitochondrial localization of estrogen receptor β. Proc Natl Acad Sci USA. (2004) 101:4130–5. 10.1073/pnas.0306948101 15024130 PMC384706

[55] Pugach EK Blenck CL Dragavon JM Langer SJ Leinwand LA. Estrogen receptor profiling and activity in cardiac myocytes. Mol Cell Endocrinol. (2016) 431:62–70. 10.1016/j.mce.2016.05.004 27164442 PMC4899180

[56] Andersson S Sundberg M Pristovsek N Ibrahim A Jonsson P Katona B Insufficient antibody validation challenges oestrogen receptor beta research. Nat Commun. (2017) 8:15840 10.1038/ncomms15840 28643774 PMC5501969

[57] Schwend T1 Gustafsson JA. False positives in MALDI-TOF detection of ERβ in mitochondria. Biochem Biophys Res Commun. (2006) 343:707–11. 10.1016/j.bbrc.2006.02.164 16563354

[58] Hutson DD Gurrala R Ogola BO Zimmerman MA Mostany R. Estrogen receptor profiles across tissues from male and female Rattus norvegicus. Biol Sex Differ. (2019) 10:4. 10.1186/s13293-019-0219-9 30635056 PMC6329134

[59] Tomicek NJ Miller-Lee JL Hunter JC Korzick DH Estrogen receptor beta does not influence ischemic tolerance in the aged female rat heart. Cardiovasc Ther. (2013) 31:32–7. 10.1111/j.1755-5922.2011.00288.x 21884022 PMC3235240

[60] Booth EA Obeid NR Lucchesi BR. Activation of estrogen receptor-α protects the *in vivo* rabbit heart from ischemia-reperfusion injury. Am J Physiol Heart Circ Physiol. (2005) 289:H2039–47. 10.1152/ajpheart.00479.2005 15994857

[61] Iorga A Umar S Ruffenach G Aryan L Li J Sharma S. Estrogen rescues heart failure through estrogen receptor Beta activation. Biol Sex Differ. (2018) 9:48. 10.1186/s13293-018-0206-6 30376877 PMC6208048

[62] Dan P Cheung JC Scriven DR Moore ED Epitope-dependent localization of estrogen receptor-α, but not -β, in en face arterial endothelium. Am J Physiol Heart Circ Physiol. (2003) 284:H1295–306. 10.1152/ajpheart.00781.2002 12531733

[63] Keung W Chan ML Ho EY Vanhoutte PM Man RY. Non-genomic activation of adenylyl cyclase and protein kinase G by 17β-estradiol in vascular smooth muscle of the rat superior mesenteric artery. Pharmacological Res. (2011) 64:509–16. 10.1016/j.phrs.2011.05.010 21641998

[64] Nakamura Y Suzuki T Miki Y Tazawa C Senzaki K Moriya T. Estrogen receptors in atherosclerotic human aorta: inhibition of human vascular smooth muscle cell proliferation by estrogens. Mol Cell Endocrinol. (2004) 219:17–26. 10.1016/j.mce.2004.02.013 15149723

[65] Lu Q Pallas DC Surks HK Baur WE Mendelsohn ME Karas RH. Striatin assembles a membrane signaling complex necessary for rapid, nongenomic activation of endothelial NO synthase by estrogen receptor α. Proc Natl Acad Sci USA. (2004) 101:17126–31. 10.1073/pnas.0407492101 15569929 PMC534607

[66] Patel VH Chen J Ramanjaneya M Karteris E Zachariades E Thomas P. G-protein coupled estrogen receptor 1 expression in rat and human heart: Protective role during ischaemic stress. Int J Mol Med. (2010) 26:193–9. 10.3892/ijmm_00000452 20596598

[67] Yu X Ma H Barman SA Liu AT Sellers M Stallone JN. Activation of G protein-coupled estrogen receptor induces endothelium-independent relaxation of coronary artery smooth muscle. Am J Physiol Endocrinol Metab. (2011) 301:E882–8. 10.1152/ajpendo.00037.2011 21791623 PMC3213995

[68] Recchia AG De Francesco EM Vivacqua A Sisci D Panno ML Andò S. The G protein-coupled receptor 30 is up-regulated by hypoxia-inducible factor-1α (HIF-1α) in breast cancer cells and cardiomyocytes. J Biol Chem. (2011) 286:10773–82. 10.1074/jbc.M110.172247 21266576 PMC3060528

[69] Murphy E Steenbergen C. Gender-based differences in mechanisms of protection in myocardial ischemia-reperfusion injury. Cardiovasc Res. (2007) 75:478–86. 10.1016/j.cardiores.2007.03.025 17466956

[70] Zhai P Eurell TE Cotthaus R Jeffery EH Bahr JM Gross DR. Effect of estrogen on global myocardial ischemia-reperfusion injury in female rats. Am J Physiol Heart Circ Physiol. (2000) 279:H2766–75. 10.1152/ajpheart.2000.279.6.H2766 11087231

[71] Wang M Crisostomo P Wairiuko GM Meldrum DR. Estrogen receptor-α mediates acute myocardial protection in females. Am J Physiol Heart Circ Physiol. (2006) 290:H2204–9. 10.1152/ajpheart.01219.2005 16415070

[72] Jeanes HL Tabor C Black D Ederveen A Gray GA Oestrogen-mediated cardioprotection following ischaemia and reperfusion is mimicked by an oestrogen receptor (ER) α agonist and unaffected by an ERβ antagonist. J Endocrinol. (2008) 197:493–501. 10.1677/JOE-08-0071 18492815 PMC2386536

[73] Zhai P Eurell TE Cooke PS Lubahn DB Gross DR. Myocardial ischemia-reperfusion injury in estrogen receptor-α knockout and wild-type mice. Am J Physiol Heart Circ Physiol. (2000) 278:H1640–7. 10.1152/ajpheart.2000.278.5.H1640 10775144

[74] Kararigas G Nguyen BT Jarry H. Estrogen modulates cardiac growth through an estrogen receptor α-dependent mechanism in healthy ovariectomized mice. Mol Cell Endocrinol. (2014) 382:909–14. 10.1016/j.mce.2013.11.011 24275180

[75] Gabel SA Walker VR London RE Steenbergen C Korach KS Murphy E. Estrogen receptor beta mediates gender differences in ischemia/reperfusion injury. J Mol Cell Cardiol. (2005) 38:289–97. 10.1016/j.yjmcc.2004.11.013 15698835

[76] Nikolic I Liu D Bell JA Collins J Steenbergen C Murphy E. Treatment with an estrogen receptor-beta-selective agonist is cardioprotective. J Mol Cell Cardiol. (2007) 42:769–80. 10.1016/j.yjmcc.2007.01.014 17362982

[77] Babiker FA Lips DJ Delvaux E Zandberg P Janssen BJ Prinzen F. Oestrogen modulates cardiac ischaemic remodelling through oestrogen receptor-specific mechanisms. Acta Physiol. (2007) 189:23–31. 10.1111/j.1748-1716.2006.01633.x 17280554

[78] Lubahn DB Moyer JS Golding TS Couse JF Korach KS Smithies O Alteration of reproductive function but not prenatal sexual development after insertional disruption of the mouse estrogen receptor gene. Proc Natl Acad Sci USA. (1993) 90:11162–6. 10.1073/pnas.90.23.11162 8248223 PMC47942

[79] Iafrati MD Karas RH Aronovitz M Kim S Sullivan TR Jr Lubahn DB. Estrogen inhibits the vascular injury response in estrogen receptor α-deficient mice. Nat Med. (1997) 3:545–8. 10.1038/nm0597-545 9142124

[80] Pendaries C Darblade B Rochaix P Krust A Chambon P Korach KS. The AF-1 activation-function of ERα may be dispensable to mediate the effect of estradiol on endothelial NO production in mice. Proc Natl Acad Sci USA. (2002) 99:2205–10. 10.1073/pnas.042688499 11854517 PMC122343

[81] Dupont S Krust A Gansmuller A Dierich A Chambon P Mark M. Effect of single and compound knockouts of estrogen receptors alpha (ERalpha) and beta (ERbeta) on mouse reproductive phenotypes. Development. (2000) 127:4277–91. 10976058 10.1242/dev.127.19.4277

[82] Brouchet L Krust A Dupont S Chambon P Bayard F Arnal JF Estradiol accelerates reendothelialization in mouse carotid artery through estrogen receptor-α but not estrogen receptor-β. Circulation. (2001) 103:423–8. 10.1161/01.CIR.103.3.423 11157695

[83] Darblade B Pendaries C Krust A Dupont S Fouque MJ Rami J Estradiol alters nitric oxide production in the mouse aorta through the α-, but not β-, estrogen receptor. Circ Res. (2002) 90:413–9. 10.1161/hh0402.105096 11884370

[84] Pare G Krust A Karas RH Dupont S Aronovitz M Chambon P. Estrogen receptor-α mediates the protective effects of estrogen against vascular injury. Circ Res. (2002) 90:1087–92. 10.1161/01.RES.0000021114.92282.FA 12039798

[85] Jesmin S Mowa CN Sultana SN Shimojo N Togashi H Iwashima Y. VEGF signaling is disrupted in the hearts of mice lacking estrogen receptor alpha. Eur J Pharmacol. (2010) 641:168–78. 10.1016/j.ejphar.2010.05.020 20639141

[86] Jakacka M Ito M Martinson F Ishikawa T Lee EJ Jameson JL. An estrogen receptor (ER)α deoxyribonucleic acid-binding domain knock-in mutation provides evidence for nonclassical ER pathway signaling *in vivo*. Mol Endocrinol. (2002) 16:2188–201. 10.1210/me.2001-0174 12351685

[87] Park CJ Zhao Z Glidewell-Kenney C Lazic M Chambon P Krust A. Genetic rescue of nonclassical ERα signaling normalizes energy balance in obese Erα-null mutant mice. J Clin Invest. (2011) 121:604–12. 10.1172/JCI41702 21245576 PMC3026715

[88] Sinkevicius KW Burdette JE Woloszyn K Hewitt SC Hamilton K Sugg SL. An estrogen receptor-α knock-in mutation provides evidence of ligand-independent signaling and allows modulation of ligand-induced pathways *in vivo*. Endocrinology. (2008) 149:2970–9. 10.1210/en.2007-1526 18339713 PMC2408815

[89] Hewitt SC O'Brien JE Jameson JL Kissling GE Korach KS Selective disruption of ERα DNA-binding activity alters uterine responsiveness to estradiol. Mol Endocrinol. (2009) 23:2111–6. 10.1210/me.2009-0356 19812388 PMC2796155

[90] Ahlbory-Dieker DL Stride BD Leder G Schkoldow J Trolenberg S Seidel H. DNA binding by estrogen receptor- α is essential for the transcriptional response to estrogen in the liver and the uterus. Mol Endocrinol. (2009) 23:1544–55. 10.1210/me.2009-0045 19574448 PMC5419145

[91] Hewitt SC Li L Grimm SA Winuthayanon W Hamilton KJ Pockette B. Novel DNA motif binding activity observed *in vivo* with an estrogen receptor α mutant mouse. Mol Endocrinol. (2014) 28:899–911. 10.1210/me.2014-1051 24713037 PMC4042070

[92] Burns KA Li Y Arao Y Petrovich RM Korach KS. Selective mutations in estrogen receptor α D-domain alters nuclear translocation and non-estrogen response element gene regulatory mechanisms. J Biol Chem. (2011) 286:12640–9. 10.1074/jbc.M110.187773 21285458 PMC3069464

[93] Stefkovich ML Arao Y Hamilton KJ Korach KS. Experimental models for evaluating non-genomic estrogen signaling. Steroids. (2018) 133:34–7. 10.1016/j.steroids.2017.11.001 29122548 PMC5864539

[94] Billon-Gales A Fontaine C Filipe C Douin-Echinard V Fouque MJ Flouriot G The transactivating function 1 of estrogen receptor α is dispensable for the vasculoprotective actions of 17 β-estradiol. Proc Natl Acad Sci USA. (2009) 106:2053–8. 10.1073/pnas.0808742106 19188600 PMC2644162

[95] Smirnova NF Fontaine C Buscato M Lupieri A Vinel A Valera MC. The activation function-1 of estrogen receptor alpha prevents arterial neointima development through a direct effect on smooth muscle cells. Circ Res. (2015) 117:770–8. 10.1161/CIRCRESAHA.115.306416 26316608 PMC4596486

[96] Billon-Gales A Krust A Fontaine C Abot A Flouriot G Toutain C Activation function 2 (AF2) of estrogen receptor-α is required for the atheroprotective action of estradiol but not to accelerate endothelial healing Proc Natl Acad Sci USA. (2011) 108:13311–6. 10.1073/pnas.1105632108 21788522 PMC3156151

[97] Guivarc'h E Buscato M Guihot AL Favre J Vessières E Grimaud L. Predominant Role of nuclear versus membrane estrogen receptor α in arterial protection: implications for estrogen receptor α modulation in cardiovascular prevention/safety. J Am Heart Assoc. (2018) 7:e008950. 10.1161/JAHA.118.008950 29959137 PMC6064913

[98] Pedram A Razandi M Kim JK O'Mahony F Lee EY Luderer U. Developmental phenotype of a membrane only estrogen receptor α (MOER) mouse J Biol Chem. (2009) 284:3488–95. 10.1074/jbc.M806249200 19054762 PMC2635032

[99] Pedram A Razandi M Lewis M Hammes S Levin ER. Membrane-localized estrogen receptor α is required for normal organ development and function. Dev Cell. (2014) 29:482–90. 10.1016/j.devcel.2014.04.016 24871949 PMC4062189

[100] Adlanmerini M Solinhac R Abot A Fabre A Raymond-Letron I Guihot AL. Mutation of the palmitoylation site of estrogen receptor α *in vivo* reveals tissue-specific roles for membrane versus nuclear actions. Proc Natl Acad Sci USA. (2014) 111:E283–90. 10.1073/pnas.1322057111 24371309 PMC3896153

[101] Moens SJB Schnitzler GR Nickerson M Guo H Ueda K Lu Q Rapid estrogen receptor signaling is essential for the protective effects of estrogen against vascular injury. Circulation. (2012) 126:1993–2004. 10.1161/CIRCULATIONAHA.112.124529 22997253 PMC3780602

[102] Ueda K Takimoto E Lu Q Liu P Fukuma N Adachi Y. Membrane-initiated estrogen receptor signaling mediates metabolic homeostasis via central activation of protein phosphatase 2A. Diabetes. (2018) 67:1524–37. 10.2337/db17-1342 29764860 PMC6054435

[103] Lu Q Schnitzler GR Ueda K Iyer LK Diomede OI Andrade T. ER Alpha rapid signaling is required for estrogen induced proliferation and migration of vascular endothelial cells. PLoS ONE. (2016) 11:e0152807. 10.1371/journal.pone.0152807 27035664 PMC4818104

[104] Mahmoodzadeh S Leber J Zhang X Jaisser F Messaoudi S Morano I. Cardiomyocyte-specific estrogen receptor alpha increases angiogenesis, lymphangiogenesis and reduces fibrosis in the female mouse heart post-myocardial infarction. J Cell Sci Ther. (2014) 5:153. 10.4172/2157-7013.1000153 24977106 PMC4070011

[105] Devanathan S Whitehead T Schweitzer GG Fettig N Kovacs A Korach KS. An animal model with a cardiomyocyte-specific deletion of estrogen receptor alpha: functional, metabolic, and differential network analysis. PLoS ONE. (2014) 9:e101900. 10.1371/journal.pone.0101900 25000186 PMC4085004

[106] Krege JH Hodgin JB Couse JF Enmark E Warner M Mahler JF. Generation and reproductive phenotypes of mice lacking estrogen receptor β. Proc Natl Acad Sci USA. (1998) 95:15677–82. 10.1073/pnas.95.26.15677 9861029 PMC28103

[107] Karas RH Hodgin JB Kwoun M Krege JH Aronovitz M Mackey W. Estrogen inhibits the vascular injury response in estrogen receptor β-deficient female mice. Proc Natl Acad Sci USA. (1999) 96:15133–6. 10.1073/pnas.96.26.15133 10611350 PMC24785

[108] Zhu Y Bian Z Lu P Karas RH Bao L Cox D. Abnormal vascular function and hypertension in mice deficient in estrogen receptor β. Science. (2002) 295:505–8. 10.1126/science.1065250 11799247

[109] Förster C Kietz S Hultenby K Warner M Gustafsson J. A Characterization of the ERβ-/– mouse heart. Proc Natl Acad Sci USA. (2004) 101:14234–9. 10.1073/pnas.0405571101 15375213 PMC521141

[110] Pelzer T Loza PA Hu K Bayer B C Dienesch C Calvillo L. Increased mortality and aggravation of heart failure in estrogen receptor-β knockout mice after myocardial infarction. Circulation. (2005) 111:1492–8. 10.1161/01.CIR.0000159262.18512.46 15781739

[111] Pedram A Razandi M O'Mahony F Lubahn D Levin ER. Estrogen receptor-beta prevents cardiac fibrosis. Mol Endocrinol. (2010) 24:2152–65. 10.1210/me.2010-0154 20810711 PMC2958752

[112] Hodgin JB Maeda N Minireview: estrogen and mouse models of atherosclerosis. Endocrinology. (2002) 143:4495–501. 10.1210/en.2002-220844 12446574

[113] Antal MC Krust A Chambon P Mark M. Sterility and absence of histopathological defects in nonreproductive organs of a mouse ERβ-null mutant. Proc Natl Acad Sci USA. (2008) 105:2433–8. 10.1073/pnas.0712029105 18268329 PMC2268154

[114] Maneix L Antonson P Humire P Rochel-Maia S Castañeda J Omoto Y. Estrogen receptorβ exon 3-deleted mouse: the importance of non-ERE pathways in ERβ signaling. Proc Natl Acad Sci USA. (2015) 112:5135–40. 10.1073/pnas.1504944112 25848008 PMC4413313

[115] Babiker FA Lips D Meyer R Delvaux E Zandberg P Janssen B. Estrogen receptor β protects the murine heart against left ventricular hypertrophy. Arterioscler Thromb Vasc Biol. (2006) 26:1524–30. 10.1161/01.ATV.0000223344.11128.23 16627800

[116] Schuster I Mahmoodzadeh S Dworatzek E Jaisser F Messaoudi S Morano I. Cardiomyocyte-specific overexpression of oestrogen receptor β improves survival and cardiac function after myocardial infarction in female and male mice. Clin Sci. (2016) 130:365–76. 10.1042/CS20150609 26608078

[117] Wang C Dehghani B Magrisso IJ Rick EA Bonhomme E Cody DB. GPR30 contributes to estrogen-induced thymic atrophy. Mol Endocrinol. (2008) 22:636–48. 10.1210/me.2007-0359 18063692 PMC2262170

[118] Mårtensson UE Salehi SA Windahl S Gomez MF Swärd K Daszkiewicz-Nilsson J. Deletion of the G protein-coupled receptor 30 impairs glucose tolerance, reduces bone growth, increases blood pressure, and eliminates estradiol-stimulated insulin release in female mice. Endocrinology. (2009) 150:687–98. 10.1210/en.2008-0623 18845638

[119] Otto C Fuchs I Kauselmann G Kern H Zevnik B Andreasen P GPR30 does not mediate estrogenic responses in reproductive organs in mice. Biol Reprod. (2009) 80:34–41. 10.1095/biolreprod.108.071175 18799753

[120] Isensee J Meoli L Zazzu V Nabzdyk C Witt H Soewarto D. Expression pattern of PR30 in LacZ reporter mice. Endocrinology. (2009) 150:1722–30. 10.1210/en.2008-1488 19095739

[121] Haas E Bhattacharya I Brailoiu E Damjanović M Brailoiu GC Gao X. Regulatory role of G protein-coupled estrogen receptor for vascular function and obesity. Circ Res. (2009) 104:288–91. 10.1161/CIRCRESAHA.108.190892 19179659 PMC2782532

[122] Meyer MR Fredette NC Howard TA Hu C Ramesh C Daniel C. G Protein-coupled estrogen receptor protects from atherosclerosis. Sci Rep. (2015) 5:13510. 10.1038/srep13510 26373621 PMC4649992

[123] Fredette NC Meyer MR Prossnitz ER. Role of GPER in estrogen-dependent nitric oxide formation and vasodilation. J Steroid Biochem Mol Biol. (2018)176:65–72. 10.1016/j.jsbmb.2017.05.006 28529128 PMC5694388

[124] Kabir ME Singh H Lu R Olde B Leeb-Lundberg LM Bopassa JC. G Protein-coupled estrogen receptor 1 mediates acute estrogen-induced cardioprotection via MEK/ERK/GSK-3β pathway after ischemia/reperfusion. PLoS ONE. (2015) 10:e0135988. 10.1371/journal.pone.0135988 26356837 PMC4565659

[125] Delbeck M Golz S Vonk R Janssen W Hucho T Isensee J. Impaired left-ventricular cardiac function in male GPR30-deficient mice. Mol Med Rep. (2011) 4:37–40. 10.3892/mmr.2010.402 21461560

[126] Wang H Sun X Chou J Lin M Ferrario CM Zapata-Sudo G. Cardiomyocyte-specific deletion of the G protein-coupled estrogen receptor (GPER) leads to left ventricular dysfunction and adverse remodeling: a sex-specific gene profiling analysis. Biochim Biophys Acta Mol Basis Dis. (2017) 1863:1870–82. 10.1016/j.bbadis.2016.10.003 27725247 PMC5385168

[127] Leinwand LA. Sex is a potent modifier of the cardiovascular system. J Clin Invest. (2003) 112:302–4. 10.1172/JCI200319429 12897194 PMC166308

[128] Yaşar P Ayaz G User SD Güpür G Muyan M. Molecular mechanism of estrogen-estrogen receptor signaling. Reprod Med Biol. (2016) 16:4–20. 10.1002/rmb2.12006 29259445 PMC5715874

[129] Acconcia F Ascenzi P Bocedi A Spisni E Tomasi V Trentalance A. Palmitoylation-dependent estrogen receptor α membrane localization: regulation by 17β-estradiol. Mol Biol Cell. (2005) 16:231–7. 10.1091/mbc.e04-07-0547 15496458 PMC539167

[130] Pedram A Razandi M Mahony FO Harvey H Harvey BJ Levin ER. Estrogen Reduces lipid content in the liver exclusively from membrane receptor signaling. Sci Signal. (2013) 6: ra36. 10.1126/scisignal.2004013 23695162

[131] Mahmoodzadeh S Dworatzek E. The Role of 17β-Estradiol and estrogen receptors in regulation of Ca2+ channels and mitochondrial function in Cardiomyocytes. Front Endocrinol. (2019) 10:310. 10.3389/fendo.2019.00310 31156557 PMC6529529

[132] Fukumoto T Tawa M Yamashita N Ohkita M Matsumura Y. Protective effects of 17beta-estradiol on post-ischemic cardiac dysfunction and norepinephrine overflow through the non-genomic estrogen receptor/nitric oxide-mediated pathway in the rat heart. Eur J Pharmacol. (2013) 699:74–80. 10.1016/j.ejphar.2012.11.042 23219795

[133] Chambliss KL Yuhanna IS Mineo C Liu P German Z Sherman TS. Estrogen receptor α and endothelial nitric oxide synthase are organized into a functional signaling module in caveolae. Circ Res. (2000) 87:E44–52. 10.1161/01.RES.87.11.e44 11090554

[134] Haynes MP Li L Sinha D Russell KS Hisamoto K Baron R. Src kinase mediates phosphatidylinositol 3-kinase/Akt-dependent rapid endothelial nitric-oxide synthase activation by estrogen. J Biol Chem. (2003) 278:2118–23. 10.1074/jbc.M210828200 12431978

[135] Simoncini T Hafezi-Moghadam A Brazil DP Ley K Chin WW Liao JK. Interaction of oestrogen receptor with the regulatory subunit of phosphatidylinositol-3-OH kinase. Nature. (2000) 407:538–41. 10.1038/35035131 11029009 PMC2670482

[136] Stefano GB Prevot V Beauvillain JC Cadet P Fimiani C Welters I. Cell-surface estrogen receptors mediate calcium-dependent nitric oxide release in human endothelia. Circulation. (2000) 101:1594–7. 10.1161/01.CIR.101.13.1594 10747354

[137] Thor D Uchizono JA Lin-Cereghino GP Rahimian R. The effect of 17 β-estradiol on intracellular calcium homeostasis in human endothelial cells. Eur J Pharmacol. (2010) 630:92–9. 10.1016/j.ejphar.2009.12.030 20044991 PMC2822064

[138] Menazza S Sun J Appachi S Chambliss KL Kim SH Aponte A. Non-nuclear estrogen receptor alpha activation in endothelium reduces cardiac ischemia-reperfusion injury in mice. J Mol Cell Cardiol. (2017) 107:41–51. 10.1016/j.yjmcc.2017.04.004 28457941 PMC5514412

[139] Ueda K Lu Q Baur W Aronovitz MJ Karas RH. Rapid estrogen receptor signaling mediates estrogen-induced inhibition of vascular smooth muscle cell proliferation. Arterioscler Thromb Vasc Biol. (2013) 33:1837–43. 10.1161/ATVBAHA.112.300752 23744991 PMC4023354

[140] Meyer MR Baretella O Prossnitz ER Barton M. Dilation of epicardial coronary arteries by the G protein-coupled estrogen receptor agonists G-1 and ICI 182,780. Pharmacology. (2010) 86:58–64. 10.1159/000315497 20639684 PMC3201835

[141] Holm A Baldetorp B Olde B Leeb-Lundberg LM Nilsson BO. The GPER1 agonist G-1 attenuates endothelial cell proliferation by inhibiting DNA synthesis and accumulating cells in the S and G2 phases of the cell cycle. J Vasc Res. (2011) 48:327–35. 10.1159/000322578 21273787

[142] Ding Q Hussain Y Chorazyczewski J Gros R Feldman RD. GPER-independent effects of estrogen in rat aortic vascular endothelial cells. Mol Cell Endocrinol. (2015) 399:60–8. 10.1016/j.mce.2014.07.023 25150623

[143] Ding Q Gros R Limbird LE Chorazyczewski J Feldman RD. Estradiol-mediated ERK phosphorylation and apoptosis in vascular smooth muscle cells requires GPR 30. Am J Physiol Cell Physiol. (2009) 297:C1178–87. 10.1152/ajpcell.00185.2009 19741198

[144] Li F Yu X Szynkarski CK Meng C Zhou B Barhoumi R. Activation of GPER induces differentiation and inhibition of coronary artery smooth muscle cell proliferation. PLoS ONE. (2013) 8:e64771. 10.1371/journal.pone.0064771 23840305 PMC3686788

[145] Yu X Stallone JN Heaps CL Han G. The activation of G protein-coupled estrogen receptor induces relaxation via cAMP as well as potentiates contraction via EGFR transactivation in porcine coronary arteries. PLoS ONE. (2018) 13:e0191418. 10.1371/journal.pone.0191418 29360846 PMC5779678

[146] Pedram A Razandi M Aitkenhead M Levin ER. Estrogen inhibits cardiomyocyte hypertrophy in vitro antagonism of calcineurin-related hypertrophy through induction of MCIP1. J Biol Chem. (2005) 280:26339–48. 10.1074/jbc.M414409200 15899894 PMC1249515

[147] Pedram A Razandi M Lubahn D Liu J Vannan M Levin ER. Estrogen inhibits cardiac hypertrophy: role of estrogen receptor-β to inhibit calcineurin. Endocrinology. (2008) 149:3361–9. 10.1210/en.2008-0133 18372323 PMC2453079

[148] Pedram A Razandi M Narayanan R Dalton JT McKinsey TA Levin ER. Estrogen regulates histone deacetylases to prevent cardiac hypertrophy. Mol Biol Cell. (2013) 24:3805–18. 10.1091/mbc.e13-08-0444 24152730 PMC3861078

[149] Hoa N Ge L Korach KS Levin ER. Estrogen receptor beta maintains expression of KLF15 to prevent cardiac myocyte hypertrophy in female rodents. Mol Cell Endocrinol. (2018) 470:240–50. 10.1016/j.mce.2017.11.004 29127073 PMC6242344

[150] Pedram A Razandi M Narayanan R Levin ER. Estrogen receptor beta signals to inhibition of cardiac fibrosis. Mol Cell Endocrinol. (2016) 434:57–68. 10.1016/j.mce.2016.06.018 27321970

[151] Dworatzek E Mahmoodzadeh S Schriever C Kusumoto K Kramer L Santos G. Sex-specific regulation of collagen I and III expression by 17β-Estradiol in cardiac fibroblasts: role of estrogen receptors. Cardiovasc Res. (2019) 115:315–27. 10.1093/cvr/cvy185 30016401 PMC6933535

[152] Lee TM Lin SZ Chang NC. Both GPER and membrane oestrogen receptor-α activation protect ventricular remodelling in 17β oestradiol-treated ovariectomized infarcted rats. J Cell Mol Med. (2014) 18:2454–65. 10.1111/jcmm.12430 25256868 PMC4302651

[153] Tong H Imahashi K Steenbergen C Murphy E. Phosphorylation of glycogen synthase kinase-3β during preconditioning through a phosphatidylinositol-3-kinase–dependent pathway is cardioprotective. Circ Res. (2002) 90:377–9. 10.1161/01.RES.0000012567.95445.55 11884365

[154] Zhang W Hou X Huang M Zeng X He X Liao Y. TDCPP protects cardiomyocytes from H2O2-induced injuries via activating PI3K/Akt/GSK3β signaling pathway. Mol Cell Biochem. (2019) 453:53–64. 10.1007/s11010-018-3431-8 30173372

[155] Pei H Wang W Zhao D Su H Su G Zhao Z. G Protein-coupled estrogen receptor 1 inhibits angiotensin II-induced cardiomyocyte hypertrophy via the regulation of PI3K-Akt-mTOR signalling and autophagy. Int J Biol Sci. (2019) 15:81–92. 10.7150/ijbs.28304 30662349 PMC6329915

[156] Li WL Xiang W Ping Y. Activation of novel estrogen receptor GPER results in inhibition of cardiocyte apoptosis and cardioprotection. Mol Med Rep. (2015) 12:2425–30. 10.3892/mmr.2015.3674 25936661 PMC4464361

[157] Feng Y Madungwe NB da Cruz Junho CV Bopassa JC. Activation of G protein-coupled oestrogen receptor 1 at the onset of reperfusion protects the myocardium against ischemia/reperfusion injury by reducing mitochondrial dysfunction and mitophagy. Br J Pharmacol. (2017) 174:4329–44. 10.1111/bph.14033 28906548 PMC5715577

[158] Rocca C Femminò S Aquila G Granieri MC De Francesco EM Pasqua T. Notch1 mediates preconditioning protection induced by GPER in normotensive and hypertensive female rat hearts. Front Physiol. (2018) 9:521. 10.3389/fphys.2018.00521 29867564 PMC5962667

[159] Senyo SE Steinhauser ML Pizzimenti CL Yang VK Cai L Wang M. Mammalian heart renewal by pre-existing cardiomyocytes. Nature. (2013) 493:433–6. 10.1038/nature11682 23222518 PMC3548046

[160] Alkass K Panula J Westman M Wu TD Guerquin-Kern JL Bergmann O. No Evidence for cardiomyocyte number expansion in preadolescent mice. Cell. (2015) 163:1026–36. 10.1016/j.cell.2015.10.035 26544945

[161] Beltrami AP Barlucchi L Torella D Baker M Limana F Chimenti S. Adult cardiac stem cells are multipotent and support myocardial regeneration. Cell. (2003) 114:763–76. 10.1016/S0092-8674(03)00687-1 14505575

[162] Bearzi C Rota M Hosoda T Tillmanns J Nascimbene A De Angelis A. Human cardiac stem cells. Proc Natl Acad Sci USA. (2007) 104:14068–73. 10.1073/pnas.0706760104 17709737 PMC1955818

[163] Leri A Rota M Pasqualini FS Goichberg P Anversa P. Origin of cardiomyocytes in the adult heart. Circ Res. (2015) 116:150–66. 10.1161/CIRCRESAHA.116.303595 25552694 PMC4283577

[164] Ellison GM Vicinanza C Smith AJ Aquila I Leone A Waring CD. Adult c-kit(pos) cardiac stem cells are necessary and sufficient for functional cardiac regeneration and repair. Cell. (2013) 154:827–42. 10.1016/j.cell.2013.07.039 23953114

[165] Vicinanza C Aquila I Scalise M Cristiano F Marino F Cianflone E Adult cardiac stem cells are multipotent and robustly myogenic: c-kit expression is necessary but not sufficient for their identification. Cell Death Differ. (2017) 24:2101–16. 10.1038/cdd.2017.130 28800128 PMC5686347

[166] Brinckmann M Kaschina E Altarche-Xifró W Curato C Timm M Grzesiak A. Estrogen receptor alpha supports cardiomyocytes indirectly through post-infarct cardiac c-kit+ cells. J Mol Cell Cardiol. (2009) 47:66–75. 10.1016/j.yjmcc.2009.03.014 19341743

[167] Chen Y Jin X Zeng Z Liu W Wang B Wang H. Estrogen-replacement therapy promotes angiogenesis after acute myocardial infarction by enhancing SDF-1 and estrogen receptor expression. Microvasc Res. (2009) 77:71–7. 10.1016/j.mvr.2008.10.003 19010336

[168] Wang L Gu H Turrentine M Wang M. Estradiol treatment promotes cardiac stem cell (CSC)-derived growth factors, thus improving CSC-mediated cardioprotection after acute ischemia/reperfusion. Surgery. (2014) 156:243–52. 10.1016/j.surg.2014.04.002 24957669

[169] Brown DA Perry JB Allen ME Sabbah HN Stauffer BL Shaikh SR. Mitochondrial function as a therapeutic target in heart failure. Nat Rev Cardiol. (2017) 14:238–50. 10.1038/nrcardio.2016.203 28004807 PMC5350035

[170] Rubinsztein DC Codogno P Levine B. Autophagy modulation as a potential therapeutic target for diverse diseases. Nat Rev Drug Discov. (2012) 11:709–30. 10.1038/nrd3802 22935804 PMC3518431

[171] Consolini AE Ragone MI Bonazzola P Colareda GA. Mitochondrial bioenergetics during ischemia and reperfusion. Adv Exp Med Biol. (2017) 982:141–67. 10.1007/978-3-319-55330-6_8 28551786

[172] Ostadal B Ostadal P. Sex-based differences in cardiac ischaemic injury and protection: therapeutic implications. Br J Pharmacol. (2014) 171:541–54. 10.1111/bph.12270 23750471 PMC3969071

[173] Stirone C Duckles SP Krause DN Procaccio V. Estrogen increases mitochondrial efficiency and reduces oxidative stress in cerebral blood vessels. Mol Pharmacol. (2005) 68:959–65. 10.1124/mol.105.014662 15994367

[174] Colom B Oliver J Roca P Garcia-Palmer FJ. Caloric restriction and gender modulate cardiac muscle mitochondrial H2O2 production and oxidative damage. Cardiovasc Res. (2007) 74:456–65. 10.1016/j.cardiores.2007.02.001 17376413

[175] Borras C Gambini J Vina J. Mitochondrial oxidant generation is involved in determining why females live longer than males. Front Biosci. (2007) 12:2120. 10.2741/2120 17127355

[176] Lagranha CJ Deschamps A Aponte A Steenbergen C Murphy E. Sex differences in the phosphorylation of mitochondrial proteins result in reduced production of reactive oxygen species and cardioprotection in females. Circ Res. (2010) 106:1681–91. 10.1161/CIRCRESAHA.109.213645 20413785 PMC3127199

[177] de Lucia C Akito Eguchi A Koch WJ. New Insights in Cardiac β-Adrenergic signaling during heart failure and aging. Front Pharmacol. (2018) 9:904. 10.3389/fphar.2018.00904 30147654 PMC6095970

[178] Ostadal B Drahota Z Houstek J Milerova M Ostadalova I Hlavackova M Developmental and sex differences in cardiac tolerance to ischemia/reperfusion injury: the role of mitochondria. Can J Physiol Pharmacol. (2019) 97:808–14. 10.1139/cjpp-2019-0060 30893574

[179] Brenner C Ventura-Clapier R Jacotot E. Mitochondria and cytoprotection. Biochem Res Int. (2012) 2012:351264. 10.1155/2012/351264 22953062 PMC3431049

[180] Imahashi K Schneider MD Steenbergen C Murphy E. Transgenic expression of Bcl-2 modulates energy metabolism, prevents cytosolic acidification during ischemia, and reduces ischemia/reperfusion injury. Circ Res. (2004) 95:734–41. 10.1161/01.RES.0000143898.67182.4c 15345651

[181] Gustafsson AB Gottlieb RA. Bcl-2 family members and apoptosis, taken to heart. Am J Physiol Cell Physiol. (2007) 292:C45–51. 10.1152/ajpcell.00229.2006 16943242

[182] Schubert C Raparelli V Westphal C Dworatzek E Petrov G Kararigas G. Reduction of apoptosis and preservation of mitochondrial integrity under ischemia/reperfusion injury is mediated by estrogen receptor β. Biol Sex Differ. (2016) 7:53. 10.1186/s13293-016-0104-8 27688871 PMC5035458

[183] Brown DA Lynch JM Armstrong CJ Caruso NM Ehlers LB Johnson MS. Susceptibility of the heart to ischaemia-reperfusion injury and exercise-induced cardioprotection are sex-dependent in the rat. J Physiol. (2005) 564:619–30. 10.1113/jphysiol.2004.081323 15718263 PMC1464442

[184] Gao J Xu D Sabat G Valdivia H Xu W Shi NQ. Disrupting KATP channels diminishes the estrogen-mediated protection in female mutant mice during ischemia-reperfusion. Clin Proteomics. (2014) 11:19. 10.1186/1559-0275-11-19 24936167 PMC4047774

[185] Liesa M Palacín M Zorzano A. Mitochondrial dynamics in mammalian health and disease. Physiol Rev. (2009) 89:799–845. 10.1152/physrev.00030.2008 19584314

[186] Zorzano A Sebastián D Segalés J Palacín M. The molecular machinery of mitochondrial fusion and fission: an opportunity for drug discovery? Curr Opin Drug Discov Devel. (2009) 12:597–606. 19736619

[187] Galloway CA Yoon Y. Mitochondrial morphology in metabolic diseases. Antioxid Redox Signal. (2013) 19:415–30. 10.1089/ars.2012.4779 22793999 PMC3700066

[188] Maneechote C Palee S Kerdphoo S Jaiwongkam T Chattipakorn SC Chattipakorn N. Balancing mitochondrial dynamics via increasing mitochondrial fusion attenuates infarct size and left ventricular dysfunction in rats with cardiac ischemia/reperfusion injury. Clin Sci. (2019) 133:497–513. 10.1042/CS20190014 30705107

[189] Chen H Chan DC. Mitochondrial dynamics in mammals. Curr Top Dev Biol. (2004) 59:119–44. 10.1016/S0070-2153(04)59005-1 14975249

[190] Zuo W Zhang S Xia CY Guo XF He WB Chen NH. Mitochondria autophagy is induced after hypoxic/ischemic stress in a Drp1 dependent manner: the role of inhibition of Drp1 in ischemic brain damage. Neuropharmacology. (2014) 86:103–15. 10.1016/j.neuropharm.2014.07.002 25018043

[191] Zepeda R Kuzmicic J Parra V Troncoso R Pennanen C Riquelme JA. Drp1 loss-of-function reduces cardiomyocyte oxygen dependence protecting the heart from ischemia-reperfusion injury. J Cardiovasc Pharmacol. (2014) 63:477–87. 10.1097/FJC.0000000000000071 24477044

[192] Ciarlo L Vona R Manganelli V Gambardella L Raggi C Marconi M. Recruitment of mitofusin 2 into “lipid rafts” drives mitochondria fusion induced by Mdivi-1. Oncotarget. (2018) 9:18869–84. 10.18632/oncotarget.24792 29721168 PMC5922362

[193] Marsboom G Toth PT Ryan JJ Hong Z Wu X Fang YH. Dynamin-related protein 1-mediated mitochondrial mitotic fission permits hyperproliferation of vascular smooth muscle cells and offers a novel therapeutic target in pulmonary hypertension. Circ Res. (2012) 110:1484–97. 10.1161/CIRCRESAHA.111.263848 22511751 PMC3539779

[194] Sharp J Farha S Park MM Comhair SA Lundgrin EL Tang WH. Coenzyme Q supplementation in pulmonary arterial hypertension. Redox Biol. (2014) 2:884–91. 10.1016/j.redox.2014.06.010 25180165 PMC4143816

[195] Liu A Philip J Vinnakota KC Van den Bergh F Tabima DM Hacker T. Estrogen maintains mitochondrial content and function in the right ventricle of rats with pulmonary hypertension. Physiol Rep. (2017) 5:e13157. 10.14814/phy2.13157 28320896 PMC5371553

[196] Sbert-Roig M Bauzá-Thorbrügge M Galmés-Pascual BM Capllonch-Amer G García-Palmer FJ Lladó I. GPER mediates the effects of 17β-estradiol in cardiac mitochondrial biogenesis and function. Mol Cell Endocrinol. (2016) 420:116–24. 10.1016/j.mce.2015.11.027 26628039

[197] Hsieh YC Yang S Choudhry MA Yu HP Rue LW 3rd Bland KI. PGC-1 upregulation via estrogen receptors: a common mechanism of salutary effects of estrogen and flutamide on heart function after trauma-hemorrhage. Am J Physiol Heart Circ Physiol. (2005) 289:H2665–72. 10.1152/ajpheart.00682.2005 16055512

[198] Zaja I Bai X Liu Y Kikuchi C Dosenovic S Yan Y. Cdk1, PKCδ and calcineurin-mediated Drp1 pathway contributes to mitochondrial fission-induced cardiomyocyte death. Biochem Biophys Res Commun. (2014) 453:710–21. 10.1016/j.bbrc.2014.09.144 25445585 PMC4312217

[199] Ikeda Y Shirakabe A Maejima Y Zhai P Sciarretta S Toli J. Endogenous Drp1 mediates mitochondrial autophagy and protects the heart against energy stress. Circ Res. (2015) 116:264–78. 10.1161/CIRCRESAHA.116.303356 25332205

[200] Pérez-Cremades D Mompeón A Vidal-Gómez X Hermenegildoand C Novella S. Role of miRNA in the regulatory mechanisms of estrogens in cardiovascular ageing. Oxidative Med Cell Longevity. (2018) 2018:6082387. 10.1155/2018/6082387 30671171 PMC6317101

[201] Zeng Y. Principles of micro-RNA production and maturation. Oncogene. (2006) 25:6156–62. 10.1038/sj.onc.1209908 17028594

[202] Florijn BW Bijkerk R van der Veer EP van Zonneveld AJ. Gender and cardiovascular disease: are sex biased microRNA networks a driving force behind heart failure with preserved ejection fraction in women? Cardiovascular Res. (2018) 114:210–25. 10.1093/cvr/cvx223 29186452

[203] Migeon R. The role of X inactivation and cellular mosaicism in women's health and sex-specific diseases. JAMA. (2006) 295:1428–33. 10.1001/jama.295.12.1428 16551715

[204] Kim VN Han J Siomi MC. Biogenesis of small RNAs in animals. Nat Rev Mol Cell Biol. (2009) 10:126–39. 10.1038/nrm2632 19165215

[205] Li P Wei J Li X Cheng Y Chen W Cui Y. 17β-estradiol enhances vascular endothelial Ets-1/miR-126–3p expression: the possible mechanism for attenuation of atherosclerosis. J Clin Endocrinol Metab. (2017) 102:594–03. 10.1210/jc.2016-2974 27870587

[206] Rippe C Blimline M Magerko K A Lawson BR LaRocca TJ Donato AJ. MicroRNA changes in human arterial endothelial cells with senescence: relation to apoptosis, eNOS and inflammation. Exp Gerontol. (2012) 47:45–51 10.1016/j.exger.2011.10.004 22037549 PMC3245334

[207] Zhang J Li SF Chen H Song JX. MiR-106b-5p inhibits tumor necrosis factor-α-induced apoptosis by targeting phosphatase and tensin homolog deleted on chromosome 10 in vascular endothelial cells. Chin Med J. (2016) 129:1406–12. 10.4103/0366-6999.183414 27270534 PMC4910362

[208] Boettger N Beetz S Kostin S Schneider J Krüger M Hein L. Acquisition of the contractile phenotype by murine arterial smooth muscle cells depends on the Mir143/145 gene cluster. J Clin Invest. (2009) 119:2634–47. 10.1172/JCI38864 19690389 PMC2735940

[209] Demolli S Doebele C Doddaballapur A Lang V Fisslthaler B Chavakis E. MicroRNA-30 mediates anti-inflammatory effects of shear stress and KLF2 via repression of angiopoietin 2. J Mol Cell Cardiol. (2015) 88:111–9. 10.1016/j.yjmcc.2015.10.009 26456066

[210] Nicholson CJ Seta F Lee S Morgan KG. MicroRNA-203 mimics age-related aortic smooth muscle dysfunction of cytoskeletal pathways. J Cell Mol Med. (2017) 21:81–95. 10.1111/jcmm.12940 27502584 PMC5192880

[211] Li H Zhou J Wei X Chen R Geng J Zheng R miR-144 and targets c-fos and cyclooxygenase-2 (COX2), modulates synthesis of PGE2 in the amnion during pregnancy and labor. Sci Rep. (2016) 6:27914 10.1038/srep27914 27297132 PMC4906292

[212] Wang X Zhu H Zhang X Liu Y Chen J Medvedovic M. Loss of the miR-144/451 cluster impairs ischaemic preconditioning-mediated cardioprotection by targeting Rac-1. Cardiovasc Res. (2012) 94:379–90. 10.1093/cvr/cvs096 22354898 PMC3331614

[213] Corcoran MP Lichtenstein AH Meydani M Dillard A Schaefer EJ Lamon-Fava S. The effect of 17β-estradiol on cholesterol content in human macrophages is influenced by the lipoprotein milieu. J Mol Endocrinol. (2011) 47:109–17. 10.1530/JME-10-0158 21830321 PMC3168528

[214] Cheng H S Sivachandran N Lau A Boudreau E Zhao JL Baltimore D MicroRNA-146 represses endothelial activation by inhibiting proinflammatory pathways. EMBO Mol Med. (2013) 5:1017–34. 10.1002/emmm.201202318 23733368 PMC3721471

[215] Bedard K Krause KH. The NOX family of ROS-generating NADPH oxidases: physiology and pathophysiology. Physiol Rev. (2007) 87:245–313. 10.1152/physrev.00044.2005 17237347

[216] Zhou J Wang KC Wu W Subramaniam S Shyy JY Chiu JJ. MicroRNA-21 targets peroxisome proliferators-activated receptor-α in an autoregulatory loop to modulate flow-induced endothelial inflammation. Proc Natl Acad Sci USA. (2011) 108:10355–60. 10.1073/pnas.1107052108 21636785 PMC3121870

[217] Thum T1 Gross C Fiedler J Fischer T Kissler S Bussen M. MicroRNA-21 contributes to myocardial disease by stimulating MAP kinase signalling in fibroblasts. Nature. (2008) 456:980–4. 10.1038/nature07511 19043405

[218] Dellago H Preschitz-Kammerhofer B Terlecki-Zaniewicz L Schreiner C Fortschegger K Chang MW. High levels of oncomiR-21 contribute to the senescence-induced growth arrest in normal human cells and its knock-down increases the replicative lifespan. Aging Cell. (2013) 12:446–58. 10.1111/acel.12069 23496142 PMC3864473

[219] Che P Liu J Shan Z Wu R Yao C Cui J. miR-125a-5p impairs endothelial cell angiogenesis in aging mice via RTEF-1 downregulation. Aging Cell. (2014) 13:926–34. 10.1111/acel.12252 25059272 PMC4331751

[220] Zhao T Li J Chen AF MicroRNA-34a induces endothelial progenitor cell senescence and impedes its angiogenesis via suppressing silent information regulator 1. Am J Physiol Endocrinol Metab. (2010) 299: E110–6. 10.1152/ajpendo.00192.2010 20424141 PMC2904051

[221] Lee CH Su SC Chiang CF Chien CY Hsu CC Yu TY. Estrogen modulates vascular smooth muscle cell function through downregulation of SIRT1. Oncotarget. (2017) 8:110039–51. 10.18632/oncotarget.22546 29299128 PMC5746363

[222] Frangogiannis NG Smith WC Entman ML. The inflammatory response in myocardial infarction. Cardiovasc Res. (2002) 53:31–47. 10.1016/S0008-6363(01)00434-5 11744011

[223] Jessup M Brozena S Heart failure New Engl J Med. (2003) 348:2007–18. 10.1056/NEJMra021498 12748317

[224] Skovgaard N Gouliaev A Aalling M Simonsen U. The role of endogenous H2S in cardiovascular physiology. Curr Pharm Biotechnol. (2011) 12:1385–93. 10.2174/138920111798280956 22309020

[225] Wang L Tang ZP Zhao W Cong BH Lu JQ Tang XL. MiR-22/Sp-1 links estrogens with the up-regulation of cystathionine -lyase in myocardium, which contributes to estrogenic cardioprotection against oxidative stress. Endocrinology. (2015) 156:2124–37. 10.1210/en.2014-1362 25825815

[226] Kehat I Davis J Tiburcy M Accornero F Saba-El-Leil MK Maillet M ERK1/2 regulate the balance between eccentric and concentric cardiac growth. Circ Res. (2011) 108:176 10.1161/CIRCRESAHA.110.231514 21127295 PMC3032171

[227] Kärkkäinen O Tuomainen T Mutikainen M Lehtonen M Ruas JL Hanhineva K Heart specific PGC-1a deletion identifies metabolome of cardiac restricted metabolic heart failure. Cardiovasc Res. (2019) 115:107–18. 10.1093/cvr/cvy155 29931052

[228] Sun LY Wang N Ban T Sun YH Han Y Sun LL. MicroRNA-23a mediates mitochondrial compromise in estrogen deficiency-induced concentric remodeling via targeting PGC-1α. J Mol and Cell Cardiol. (2014) 75:1–11. 10.1016/j.yjmcc.2014.06.012 24984145

[229] Zhang Y Wang R Du W Wang S Yang L Pan Z. Downregulation of miR-151–5p contributes to increased susceptibility to arhythmogenesis during myocardial infarction with estrogen deprivation. PLoS ONE. (2013) 8:e72985. 10.1371/journal.pone.0072985 24039836 PMC3767733

[230] Tang ZP Zhao W Du JK Ni X Zhu XY Lu JQ. Mir-494 contributes to estrogen protection of cardiomyocytes against oxidative stress via targeting (nF-κB) repressing factor. Front Endocrinol. (2018) 9:215. 10.3389/fendo.2018.00215 29867756 PMC5960695

